# Stromal NADH supplied by PHOSPHOGLYCERATE DEHYDROGENASE3 is crucial for photosynthetic performance

**DOI:** 10.1093/plphys/kiaa117

**Published:** 2021-01-13

**Authors:** Ricarda Höhner, Philip M Day, Sandra E Zimmermann, Laura S Lopez, Moritz Krämer, Patrick Giavalisco, Viviana Correa Galvis, Ute Armbruster, Mark Aurel Schöttler, Peter Jahns, Stephan Krueger, Hans-Henning Kunz

**Affiliations:** 1 School of Biological Sciences, Washington State University, Pullman, WA 99164-4236, USA; 2 Biocenter University of Cologne, Institute for Plant Science, Cologne 50674, Germany; 3 Max Planck Institute for Biology of Ageing, Cologne 50933, Germany; 4 Max Planck Institute of Molecular Plant Physiology, Wissenschaftspark Golm, Potsdam 14476, Germany; 5 Plant Biochemistry, Heinrich-Heine-University Düsseldorf, Düsseldorf D-40225, Germany

## Abstract

During photosynthesis, electrons travel from light-excited chlorophyll molecules along the electron transport chain to the final electron acceptor nicotinamide adenine dinucleotide phosphate (NADP) to form NADPH, which fuels the Calvin–Benson–Bassham cycle (CBBC). To allow photosynthetic reactions to occur flawlessly, a constant resupply of the acceptor NADP is mandatory. Several known stromal mechanisms aid in balancing the redox poise, but none of them utilizes the structurally highly similar coenzyme NAD(H). Using Arabidopsis (*Arabidopsis thaliana*) as a C_3_-model, we describe a pathway that employs the stromal enzyme PHOSPHOGLYCERATE DEHYDROGENASE 3 (PGDH3). We showed that PGDH3 exerts high NAD(H)-specificity and is active in photosynthesizing chloroplasts. PGDH3 withdrew its substrate 3-PGA directly from the CBBC. As a result, electrons become diverted from NADPH via the CBBC into the separate NADH redox pool. *pgdh3* loss-of-function mutants revealed an overreduced NADP(H) redox pool but a more oxidized plastid NAD(H) pool compared to wild-type plants. As a result, photosystem I acceptor side limitation increased in *pgdh3*. Furthermore, *pgdh3* plants displayed delayed CBBC activation, changes in nonphotochemical quenching, and altered proton motive force partitioning. Our fluctuating light-stress phenotyping data showed progressing photosystem II damage in *pgdh3* mutants, emphasizing the significance of PGDH3 for plant performance under natural light environments. In summary, this study reveals an NAD(H)-specific mechanism in the stroma that aids in balancing the chloroplast redox poise. Consequently, the stromal NAD(H) pool may provide a promising target to manipulate plant photosynthesis.

## Teaser

PHOSPHOGLYCERATE DEHYDROGENASE 3, an oxidoreductase in leaf chloroplasts with strong preference to reduce the stromal NAD(H) instead of the NADP(H) pool, is required for full photosynthetic capacity.

## Introduction

Cellular life utilizes two closely related nicotinamide adenine dinucleotide coenzymes, nicotinamide adenine dinucleotide NAD(H) and nicotinamide adenine dinucleotide phosphate NADP(H), to assist in a variety of redox reactions. The two compounds are structurally very similar but fulfill unique roles in biochemical pathways. NADP is preferably used in anabolic processes while catabolic reactions mostly rely on NAD (Takase et al., 2014). Plant leaf cells harbor chloroplasts, a specialized type of plastid, in which photosynthesis takes place. Photosynthesis facilitates the conversion of light energy and CO_2_ into chemically stored energy. During this process, electrons extracted from water molecules travel along the linear electron transport chain from photosystem II (PSII) to PSI and ferredoxin. Finally, the enzyme ferredoxin-NADP reductase (FNR) assists in temporally storing the electrons as redox power in the form of NADPH. Photosynthetic electron transport also generates a proton motive force (pmf) across the thylakoid membrane, which drives ATP synthesis by the chloroplast ATP synthase. Subsequently, ATP and NADPH are used to fuel the Calvin–Benson–Bassham cycle (CBBC) and other reactions. In the absence of light or during stress, the oxidative pentose phosphate pathway supplies NADPH to keep biosynthetic pathways running (Kruger and von Schaewen, 2003; Sharkey and Weise, 2015).

Plastids can neither take up nor export NADP. Additionally, no envelope membrane uptake mechanism exists for the reduced coenzymes NAD(P)H. Thus, the replenishing of the stromal nicotinamide adenine dinucleotide pool relies on the import of NAD from its production site, the cytosol (Hashida and Kawai-Yamada, 2019). The carrier(s) facilitating the plastid NAD transport remain unknown (de Souza Chaves et al., 2019). Upon uptake, the stromal ATP-dependent NAD kinase 2 (NADK2) catalyzes the conversion of NAD to NADP (Chai et al., 2005).

A buildup of reduced NADPH coenzymes results in an overreduced stroma, which hampers light-dependent reactions and triggers production of reactive oxygen species that can readily cause cell damage (Alric and Johnson, 2017). To avoid this unfavorable situation, chloroplasts mainly use three mechanisms to release reducing equivalents into the cytosol (reviewed in Dietz et al., 2016). First, the triose phosphate/phosphate translocator (TPT) can shuttle triose phosphate into the cytosol (Schneider et al., 2002; Walters et al., 2004). Triose phosphate formation is catalyzed by the action of the NADP-glyceraldehyde 3-phosphate dehydrogenase (GAPDH) and triose-phosphate isomerase as part of the reduction phase of the CBBC. Second, two independent malate valves exist. Each one consists of either an NADP- (Scheibe, 1987) or an NAD-specific malate dehydrogenase (MDH; Berkemeyer et al., 1998) accompanied by inner envelope oxaloacetate/malate exchanger(s) (Kinoshita et al., 2011). Once malate has been exported, electrons are released by cytosolic MDH enzymes (reviewed in Selinski and Scheibe, 2019). In summary, the described mechanisms function to ensure sufficient supply of oxidized, electron-accepting coenzymes in the chloroplast stroma to maintain the flow of electrons from the photosynthetic electron transport chain. As a backup, chloroplasts possess alternative electron transport pathways, such as cyclic electron flow around PSI and a plastid terminal oxidase (reviewed in Alric and Johnson, 2017). Additionally, plants can reversibly downregulate linear electron transport by “photosynthetic control” of plastoquinol oxidation at the cytochrome b_6_f complex, which usually is the rate-limiting step of photosynthesis (reviewed by Schöttler et al., 2014).

Generally, dehydrogenases, the class of electron transferring enzymes, exhibit high specificity toward either NAD(H) or NADP(H). This feature allows the simultaneous maintenance of two separate redox coenzyme factor pools in cells and organelles (Cahn et al., 2017). The majority of redox reactions in the stroma employ NADP(H), FAD(H), and thioredoxin (Geigenberger and Fernie, 2014). Nevertheless, a few NAD(H)-dependent reactions have been reported but mostly occur in plastids of heterotrophic tissue where catabolic reaction pathways dominate (Selinski and Scheibe, 2019). More recent studies revealed that at least two NAD(H)-dependent enzymes, NAD-MDH and enoyl-ACP reductase, are also active in photoautotrophic leaf chloroplasts. Respective mutant lines with reduced enzyme activities display strong leaf phenotypes (Beeler et al., 2014; Wu et al., 2015). However, because both reactions oxidize NADH *in vivo* it is unknown which reaction(s) initially generate reduced stromal NADH (Zhao et al., 2020).

In this study, we set out to identify enzymatic reactions in the chloroplast stroma that function as a source of reduced NADH throughout the day. In doing so, we aimed to gain initial insights into the physiological relevance of the NAD(H) pool in illuminated autotrophic plastids especially with regard to C_3_ photosynthesis. Evidence for at least two distinct NADH-yielding reactions in chloroplasts can be found in the literature: (1) Pyruvate dehydrogenase activity was measured in isolated pea (*Pisum sativum*) chloroplasts (Camp and Randall, 1985). The NAD-specific dehydrogenase is part of a large enzyme supercomplex, which catalyzes the early steps of fatty acid synthesis (Blume et al., 2013). The pyruvate dehydrogenase complex (PDC) has been described as an example for substrate channeling (Roughan, 1997): Substrates and cofactors are passed on directly to enzymes catalyzing follow-up reactions to avoid diffusion limitation (Sweetlove and Fernie, 2018). Pyruvate dehydrogenase likely provides NADH directly to enoyl-ACP reductase (Camp and Randall, 1985; Slabas et al., 1986) and was therefore not considered an ideal starting point for this study. (2) At least one of the three plastid phosphoglycerate dehydrogenase (PGDH) isoforms in Arabidopsis (*Arabidopsis thaliana*; Benstein et al., 2013; Toujani et al., 2013) might be active in autotrophic tissue because an earlier study reported PGDH activity in leaf extracts from the C_3_ plant spinach (*Spinacia oleracea*; Larsson and Albertsson, 1979). Thus far, only mutants defective in *PGDH1* have revealed a phenotype that proved the importance of this particular isoform in heterotrophic tissues (Benstein et al., 2013; Cascales-Miñana et al., 2013). However, studies on *pgdh2* and *pgdh3* loss-of-function mutants did not unveil any quantifiable changes from wild-type (WT) plants (Benstein et al., 2013; Toujani et al., 2013).

In this study we determined the coenzyme specificity of the plastid PGDHs and identified photosynthesis-related phenotypes in Arabidopsis *pgdh3* loss-of-function mutants.

## Results

### The three plastid PGDHs reveal strong coenzyme-specificity for NAD

**Figure 1:**
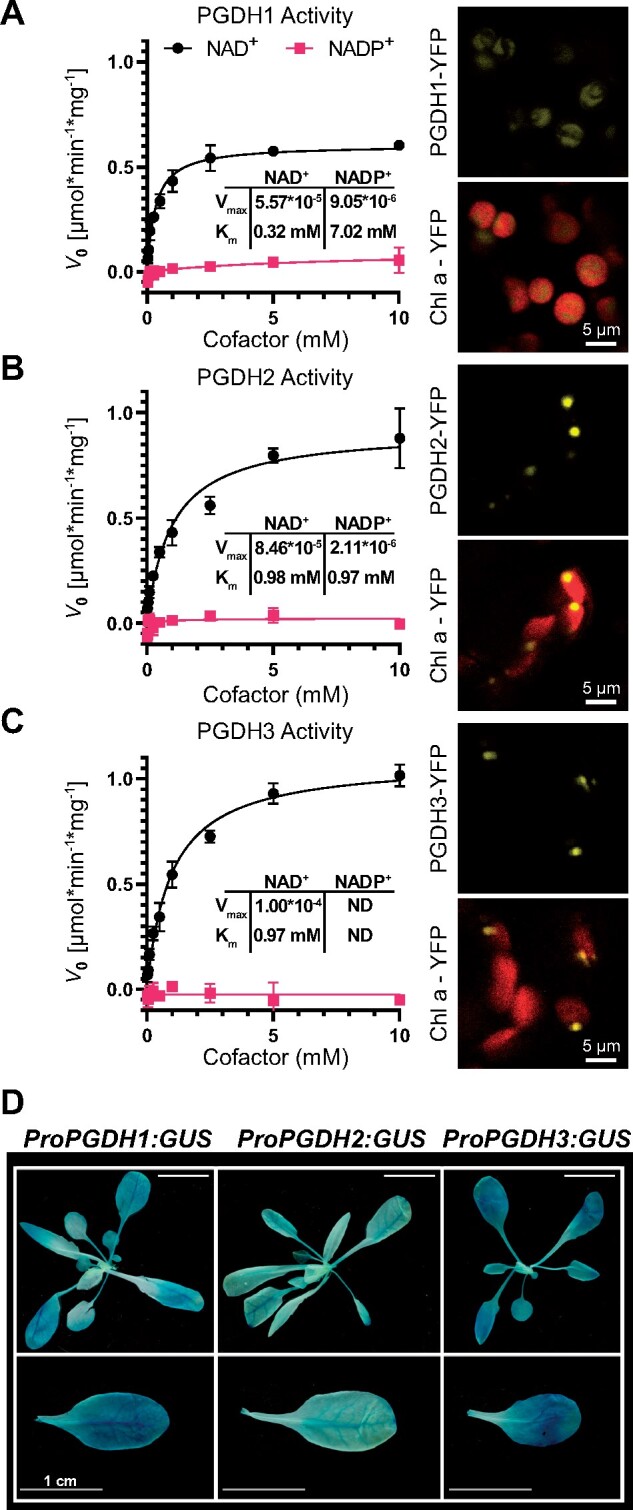
Co-factor specificity studies reveal PGDH3 as highly NAD-dependent and the most strongly expressed isoform in the mesophyll leaf tissue. A–C (left), Phosphoglycerate dehydrogenase activity at various concentrations of NAD (black squares) or NADP (red circles) from total enzyme extracts of leaves transiently expressing *PGDH1-YFP* (A), *PGDH2-YFP* (B), and *PGDH3-YFP* (C) (±sd, *n *=* *3 per isoform). A–C (right), Confocal laser scanning micrographs of leaf cells transiently expressing YFP fusions. YFP signal (yellow) is shown on top. YFP signal overlaid on chlorophyll autofluorescence (red) is shown below (scale bars = 5 *μ*m). D, GUS stain for all three PGDH isoforms shows that *PGDH3* is primarily expressed in leaf mesophyll cells (scale bars = 1 cm).

Independent studies have confirmed the NAD-dependent dehydrogenase activity of recombinant PGDHs using 3-phosphoglycerate (3-PGA) as a substrate (Larsson and Albertsson, 1979; Ho et al., 1999; Benstein et al., 2013; Okamura and Hirai, 2017). However, it was never examined to what degree plastid PGDHs exert dual coenzyme specificity for NAD and NADP as it is the case among glucose-6-phosphate (G6P) dehydrogenase enzymes (Levy, 1979; Olavarría et al., 2012).cDNAs from all three Arabidopsis PGDH isoforms were cloned upstream of a C-terminal yellow fluorescent protein (YFP) fusion protein and transiently expressed in *Nicotiana benthamiana* leaf tissue under the control of the AtUBQ10 promotor (Grefen et al., 2010). Five days after injection robust expression was verified by confocal microscopy (Figure 1, A–C inlets). While all three isoforms were localized in the chloroplast, PGDH2 and PGDH3 appeared in punctuated spots. Total leaf proteins were extracted and the coenzyme specificity for each Arabidopsis PGDH isoform was determined by increasing the NAD or NADP concentration, respectively, from 0.025 to 10 mM. In all reactions, the enzymes were kept in reducing buffers simulating the stromal daytime conditions with substrate level saturated at 5-mM 3-PGA. The reaction plots in Figure 1, A–C show that all three isoforms exerted dramatically higher activity in the presence of NAD compared to NADP. The NAD coenzyme preference for all PGDH isoforms was further corroborated by deducing *V*_max_ and *K*_m_ values (Figure 1, A–C). The two most closely related isoforms, PGDH1 and PGDH3, both showed much higher binding affinity (lower *K*_m_) to NAD over NADP. As an expression control and to determine the endogenous background activity in *N. benthamiana*, a chloroplast stromal targeted YFP was employed (Mehlmer et al., 2012). The total endogenous NtPGDH leaf activity was <12% of the activity in leaves transiently overexpressing one of the three PGDH isoforms from Arabidopsis (Supplemental Figure 1A).

To identify the main isoform responsible for the reported PGDH activity in leaf cells (Larsson and Albertsson, 1979), we verified earlier promoter GUS studies (Benstein et al., 2013). *pPGDH3::GUS* plants displayed the strongest dark blue GUS stain in the leaf mesophyll, indicative of robust gene expression (Figure 1D). *PGDH1* also showed clear expression in leaves, but its GUS signal was more restricted to the vascular tissue. *pPGDH2::GUS* signal was low in the aerial tissue and strongly restricted to the leaf veins. The results are in line with publicly available *PGDH* gene expression data (Winter et al., 2007; Supplemental Figure 1, B and C). In summary, our results reveal PGDH3 as a NAD-dependent enzyme with strong expression in the leaf tissue.

### Loss of PGDH3 results in elevated transient nonphotochemical quenching under nonsaturating light conditions

**Figure 2:**
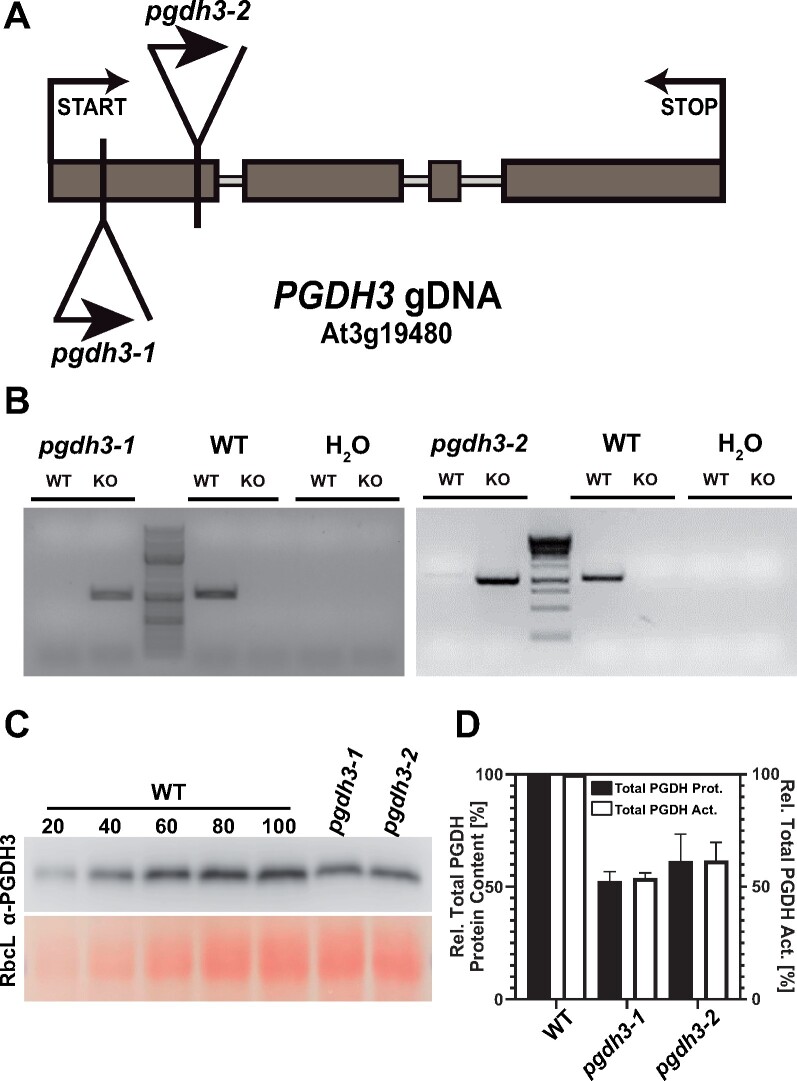
Molecular characterization of *pgdh3* T-DNA insertion lines confirms diminished PGDH amounts and enzyme activity in leaves. A, Genomic locus map of the *PGDH3* locus*.* Both T-DNA insertions are located in the first exon. Exons = dark gray boxes. B, Genotyping by PCR confirmation of homozygous insertions and gene disruption in *pgdh3-1* and *pgdh3-2*. C, Above, immunoblotting of total protein extracted from WT and *pgdh3* mutant plant leaf tissue using α-PGDH3. The WT 100, *pgdh3-1*, and *pgdh3-2* lanes contain the equivalent of 2.5 mg fresh mass of leaf tissue. WT 20–80 lanes contain the corresponding percentage of the WT sample. Below, Ponceau red stain of the region of the blot occupied by the large subunit of rubisco (RbcL). D, Bar graph showing a drop of 46.2% in total leaf PGDH activity in *pgdh3-1* (54% total PGDH protein content) and 38.3% in *pgdh3-2* (60% total PGDH protein content) relative to WT (±sd, *n *≥* *3 per genotype).

To allow for detailed physiological plant studies, we initially confirmed the homozygous genotype of previously isolated independent T-DNA insertion lines *pgdh3-1* and *pgdh3-2* in the *PGDH3* (At3g19480) locus (Figure 2, A and B;  Toujani et al., 2013). In the same study, no residual *PGDH3* mRNA was detected in either homozygous line (Toujani et al., 2013). We generated a polyclonal PGDH antibody (α-PGDH3) using recombinant AtPGDH3 protein as the antigen. A specificity test confirmed that the α-PGDH3 immunoglobulin recognized all three Arabidopsis PGDH isoforms (Supplemental Figure 2, A–C). Next up, we probed leaf protein extracts from WT, *pgdh3-1*, and *pgdh3-2* to determine total PGDH protein contents by immunoblotting (Figure 2C). With the help of a standard curve from WT extracts, residual PGDH contents in mutant plants were determined as 54% in *pgdh3-1* and 60% in *pgdh3-2* (Figure 2D). Additionally, we assayed total PGDH activity in the aerial tissue. In line with the immunoblotting results, we documented a 46.2% and 38.3% decrease of total NAD-dependent PGDH activity in *pgdh3-1* and *pgdh3-2*, respectively (Figure 2D). Therefore, PGDH3 activity closely follows changes in enzyme content.

**Figure 3:**
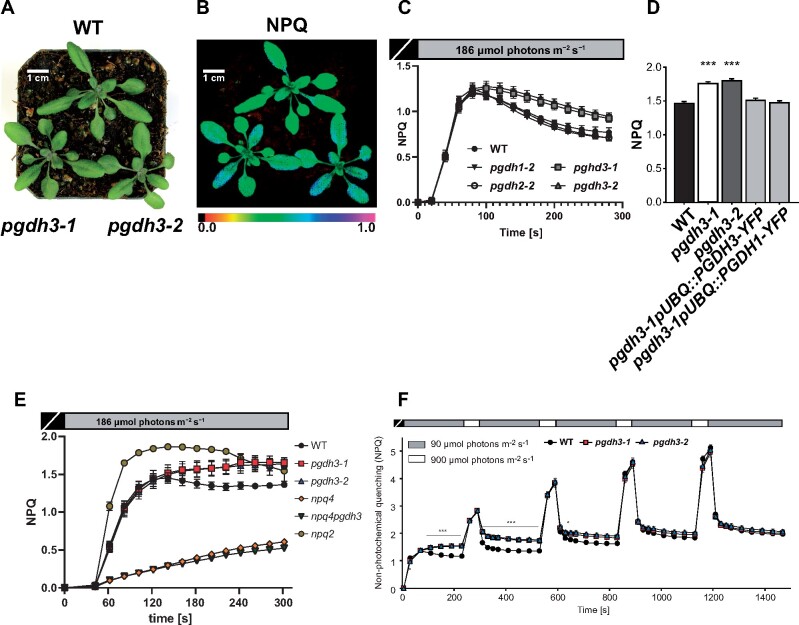
Phenotypic studies of *pgdh3* mutants reveal alterations in photosynthetic performance. A, 21-d-old WT and *pgdh3* mutant plants grown in standard long-day conditions. Mutants do not exert a visual phenotype but (B) show higher NPQ than WT controls. C, Only the loss of the PGDH3 isoform causes increased transient NPQ levels (±se, *n *≥* *13 per genotype). D, Overexpression of either *PGDH1* or *PGDH3* rescues the NPQ mutant phenotype (±se, *n *≥* *3 per genotype). E, Comparing transient NPQ kinetics (actinic light intensity: 186-*μ*mol photons m^−2^ s^−1^) of 21-d-old WT and *pgdh3* mutant plants shows that in the absence of *PsbS* transient NPQ is not different between *npq4* and *npq4pgdh3* loss of function mutants (±se, *n *=* *5 per genotype). F, WT and *pgdh3* mutant plants grown under ambient conditions were exposed to fluctuating light (LL, 4 min 90-*μ*mol photons m^−2^ s^−1^; HL, 1 min 900-*μ*mol photons m^−2^ s^−1^) using the Imaging PAM, and chlorophyll-*a* fluorescence was recorded. Asterisks above and below traces mark when NPQ was significantly increased in both *pgdh3* mutant alleles as compared to WT during LL phases (*0.01 < *p* < 0.05, ***0.001 < *p*; one-way ANOVA and Tukey pairwise comparison). Traces represent averages extracted from one representative experiment (±se, *n *=* *9 per genotype). The experiment was repeated three times and the same results replicated each time.

When the two loss-of-function *pgdh3* mutants were grown in normal long day conditions (16-h/8-h day–night cycle, 150 µmol photons m^−2^ s^−1^), no differences from WT controls in appearance or growth behavior were observed (Figure 3A). The leaf pigment composition was barely altered from WT with only a slightly lower total chlorophyll content found in *pgdh3-1* but not in *pgdh3-2* (Table 1). 77K fluorescence emission spectra on WT and both *pgdh3* plants (harvested in the middle of the 16h light period, 150 µmol photons m^−2^ s^−1^) did not indicate mutant-specific changes in the light-harvesting complex distribution between PSI or PSII (Supplemental Figure 3A). All  light harvesting complexes (LHCs) were efficiently coupled to their reaction centers, because no emission bands indicative of free LHCII or LHCI could be observed. Next, pulse-amplitude modulated (PAM) chlorophyll-*a* fluorescence measurements were employed to determine basic photosynthesis parameters by means of an induction curve at nonsaturating light conditions (actinic light 186 *µ*mol photons m^−2^ s^−1^). Interestingly, under these conditions, both *pgdh3* mutant lines revealed prolonged transient nonphotochemical quenching (NPQ) not reaching steady-state level within the 5 min of the recording (Figure 3, B and C). This effect was specific to the lack of PGDH3 function as the loss of the two other isoforms did not result in high NPQ (Figure 3C). WT NPQ levels were restored when either *PGDH1-YFP* or *PGDH3-YFP* were overexpressed under the control of the *AtUBQ10* promotor in the *pgdh3-1* mutant background (Figure 3D; Supplemental Figure 3, B–D).

**Table 1:** Leaf pigment composition in WT and *pgdh3* plants

	Pigment content in pmol*mg^−1^ FW					
Genotype	Nx	Lut	Car	VAZ	Chl (a + b)	Chl a/b	
WT	40 ± 5^a^	143 ± 18^a^	92 ± 10^a^	43 ± 10^a^	1368 ± 129^a^	3.07 ± 0.07^a^	
*pgdh3-1*	36 ± 4^a^	139 ± 18^a^	84 ± 7^a^	45 ± 7^a,b^	1185 ± 123^b^	3.08 ± 0.06^a,b^	
*pgdh3-2*	39 ± 5^a^	140 ± 14^a^	85 ± 10^a^	38 ± 5^a,c^	1317 ± 139^a,b^	3.02 ± 0.05^a,c^	

Total chlorophyll levels were slightly but significantly decreased in *pgdh3-1* but not in *pgdh3-2*. Chlorophyll (Chl) content and Chl a/b ratio was the same among all genotypes. No changes were found for neoxanthin (Nx), lutein (Lut), β-carotene (Car), and VAZ (sum of violaxanthin, antheraxanthin, zeaxanthin) in WT and *pgdh3* plants. Values represent means ± se of eight independent measurements (Student’s *t* test, *p* < 0.05).

Prolonged transient NPQ signatures can indicate a delayed CBBC activation (Kalituho et al., 2007; Okegawa and Motohashi, 2015; Thormählen et al., 2017). NPQ is composed of several different components of which the pH-dependent factor qE represents the main contributor (Müller et al., 2001). The transient NPQ at nonsaturating light initially shows a characteristic rapid increase, driven by the buildup of a trans-thylakoid proton gradient, followed by a clear drop in NPQ (∼100 s). This NPQ drop marks the activation of the CBBC and concomitant ATP and NADPH consumption. As the proton gradient decreases so does the transient NPQ and qE (Kalituho et al., 2007). In plants, qE is trigged by the PsbS (NPQ4) protein (Niyogi et al., 2004). We isolated *pgdh3npq4* double mutants and found that the elevated transient NPQ was abolished, i.e. NPQ values were not different from *npq4-1* single mutants (Figure 3E). This indicates that the main contributor to the observed elevated NPQ in *pgdh3* loss-of-function mutants is indeed low luminal pH-triggered qE. In line with the hypothesis of a delayed CBBC activation in *pgdh3*, we found that when the nonsaturating light induction time was extended to 30 min, steady-state NPQ levels in *pgdh3-1* mutants were very close to WT NPQ values without quite reaching the same low level in *pgdh3-2* (Supplemental Figure 4A). At saturating light (925 µmol photons m^−2^ s^−1^), when proton flux into the thylakoid exceeds proton consumption by ATP synthesis (Kalituho et al., 2007), differences in transient NPQ between WT and mutants were abolished. A similar effect was seen in short-term dynamic light regimes [low light (LL), 4 min 90 μmol photons m^−2^ s^−1^; high light (HL), 1 min 900-*μ*mol photons m^−2^ s^−1^] (Figure 3F). NPQ in both *pgdh3* lines only increased during the LL phases but reproduced WT values during HL periods. As expected, differences between WT and mutant NPQ vanished with every light cycle and were entirely gone from the fourth repetition when the CBBC was fully activated in mutants. Lastly, we probed the xanthophyll cycle in *pgdh3* mutants, i.e. the light-dependent (15 min light phase at 900 *µ*mol photons m^−2^ s^−1^, white light) and reversible conversion of violaxanthin (Vx) to zeaxanthin (Zx; 30-min dark phase). No significant differences were observed in the light-dependent de-epoxidation kinetic. However, both *pgdh3* loss-of-function lines remained in a prolonged de-epoxidation state after the actinic light was turned off, which may indicate changes in the stromal redox poise (Supplemental Figure 4B).

**Figure 4:**
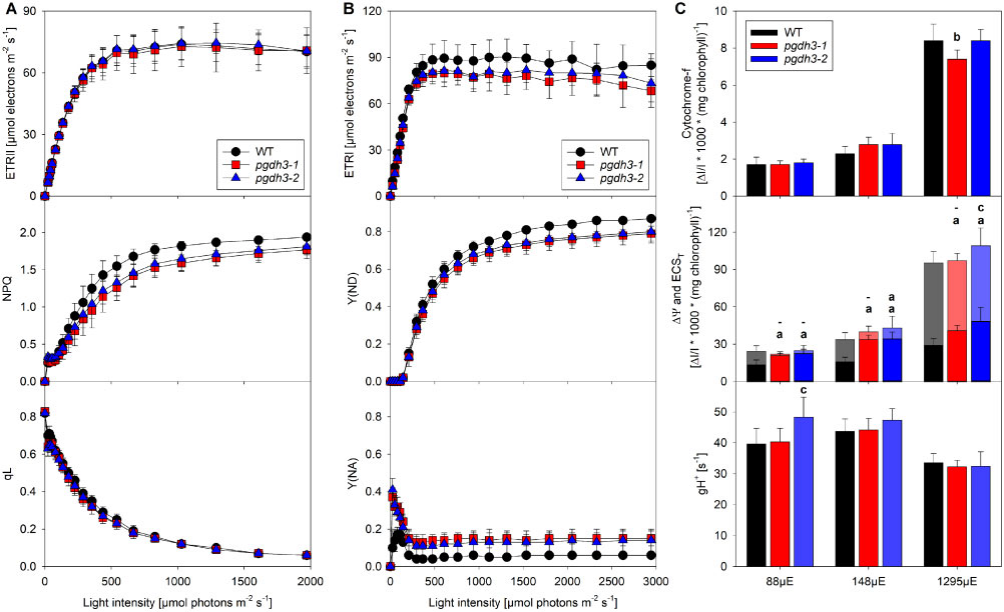
*pgdh3* mutants show pronounced acceptor side limitation and altered pmf partitioning. Chlorophyll-*a* fluorescence was recorded with a slow light response curve on dark-adapted WT and *pgdh3* plants. A, No changes were found in ETRII or in the fraction of oxidized Q_A_ (qL). NPQ in mutants was lower at light intensities above 400-*μ*mol photons m^−2^ s^−1^. B, P_700_ parameters were measured on plants taken directly from the controlled environment chamber and deconvoluted from difference absorbance signals arising from plastocyanin. The electron transfer rates of PSI (ETRI) in both *pgdh3* lines were slightly decreased. PSI was by trend less donor-side limited (Y(ND)) in *pgdh3* when actinic light exceeded 500-*μ*mol photons m^−2^ s^−1^. Independent of the light intensity *pgdh3* revealed significantly stronger acceptor side limitation (Y(NA)). C, Neither the cytochrome-f oxidation level nor the proton conductivity of the thylakoid membrane (*g*H^+^) were altered at three different light intensities. While the total pmf size (ECS_T_) was not different among genotypes (transparent bars), both *pgdh3* mutant lines showed much stronger ΔΨ partitioning at low (88) and ambient (144-*μ*mol photons m^−2^ s^−1^) actinic light (solid bars). Data represent mean values (±sd) of nine independent samples. Letters indicate significantly different values between WT and the individual *pgdh3* mutant lines as determined by ANOVA (*p* < 0.05), with (a) indicating significant differences from the WT, (b) indicating significant differences from both WT and *pgdh3-2*, and (c) indicating significant differences from both WT and *pgdh3-1*. For the pmf panel, the upper row indicates significant differences of ECS_T_, while the lower row indicates significant differences of ΔΨ. To clarify which letter refers to ECS_T_ or ΔΨ, in this panel, also nonsignificant statistical results are indicated by a horizontal line.

In summary, the elevated and prolonged transient NPQ at low, nonsaturating light conditions hints at a delayed CBBC activation in mesophyll cells in the absence of PGDH3 function.

### Light response curves of photosynthesis reveal acceptor side limitation of PSI and changes in proton motive force partitioning in pgdh3 mutants

More information on how decreased chloroplast PGDH activity affects the light-dependent reactions was gathered through recording several parameters related to PSII and PSI activity by means of light response curves (0–2,000 *µ*mol photons m^−2^ s^−1^) of chlorophyll-*a* fluorescence parameters (Figure 4A) and PSI-related parameters (Figure 4B). Measurements were performed at ambient CO_2_ concentration (400 ppm). While dark-adapted leaves were used for the chlorophyll-*a* fluorescence measurements, for the PSI measurements, pre-illuminated leaves were employed, and PSI-related signals were deconvoluted from absorbance changes of plastocyanin. Under light-limited conditions, the light intensity was slowly increased, to avoid an overlap of the light response curve with the transient NPQ effects occurring during photosynthetic induction (Figure 3, C–E). As shown in Figure 4, A B, both independent *pgdh3* loss-of-function lines revealed normal electron transfer rates (ETRs) of PSII but slightly decreased PSI ETR when actinic light intensities exceeded 400 *µ*mol photons m^−2^ s^−1^. No differences were found in the number of open PSII reaction centers (qL), i.e. the PSII acceptor side was similarly reduced across all lines. Interestingly, as the light intensities surpassed 200 *µ*mol photons m^−2^ s^−1^, NPQ in *pgdh3* mutants did not increase to the same degree as in WT (Figure 4A).

As mentioned before, PSI ETRs were slightly lower in mutants (Figure 4B). A detailed analysis of the limitations at the PSI acceptor and donor side (Y(NA) and Y(ND), respectively) revealed that in the mutants, the donor-side limitation of PSI was less pronounced. However, this did not result in increased electron transport, because PSI was more strongly limited on its acceptor side in both *pgdh3* mutant lines. This effect was especially apparent at lower light intensities and further indicates problems in the mutant’s CBBC.

Finally, the redox state of cytochrome-f, the total light-induced pmf across the thylakoid membrane, and its partitioning into ΔΨ and ΔpH were determined at three different light intensities by interrupting steady-state photosynthesis with a short interval of darkness (Figure 4C). Furthermore, the proton conductivity of the thylakoid membrane (*g*H^+^) was measured as a proxy for ATPase activity: When chloroplast ATP synthase is fully activated in the light, its activity is the predominant determinant for the rapid dark-interval relaxation of the light-induced pmf (reviewed by Baker et al., 2007). In parallel to the decay of the light-induced pmf, cytochrome-f, which becomes increasingly oxidized at higher actinic light intensities, is rapidly reduced by electrons stored in the plastoquinone pool. All signal amplitudes were normalized to the chlorophyll content of the measured leaf area. Similar amplitudes of the three difference transmittance signals arising from cytochrome-f in saturating light (1,295 *µ*E m^−2^ s^−1^), when cytochrome-f is fully oxidized, indicated largely unaltered contents of the rate-limiting cytochrome b_6_f complex. This is in agreement with the unaltered capacity of linear electron transport and the similar redox state of the PSII acceptor side (Figure 4A). Also, at intermediate (148 *µ*E m^−2^ s^−1^) and low light intensities (88 *µ*E m^−2^ s^−1^), the amount of oxidized cytochrome-f did not differ much between the WT and the mutants. This argues against a stronger thylakoid lumen acidification in the mutants under steady-state conditions. Stronger thylakoid acidification slows plastoquinol oxidation at the cytochrome b_6_f complex and therefore would result in a higher oxidation state of cytochrome-f. Indeed, measurements of the total light-induced electrochromic shift signal (ECS_T_) suggest very similar total pmf values across the thylakoid membrane in the WT and the mutants at all three light intensities. Exemplary non-normalized dark interval relaxation kinetics of the ECS at all three actinic light intensities are shown in Supplemental Figure 4C. Remarkably, the slow inverted phase of the ECS dark-interval relaxation kinetic (ECS_inv_), which reflects the fraction of the pmf stored as ΔpH (as indicated for the WT signal at the highest actinic light intensity of 1,295 *µ*E m^−2^ s^−1^, Supplemental Figure 4C) revealed drastic differences between the WT and both mutant lines. In both *pgdh3* mutants, pmf partitioning clearly shifted towards ΔΨ, at the expense of ΔpH. Especially at lower (88 *µ*mol photons m^−2^ s^−1^) to middle (148 *µ*mol photons m^−2^ s^−1^) actinic light intensities, the pmf in *pgdh3* was almost entirely stored as ΔΨ, with the fraction being much larger than in WT under the same conditions.

In summary, the collected spectroscopy data indicate that lower stromal phosphoglycerate dehydrogenase activity in *pgdh3* lines results in a significant PSI acceptor side limitation and changes in pmf partitioning.

### pgdh3 loss-of-function mutants have lower CO_2_ fixation rates at high Ci level

At low, nonsaturating light intensities chlorophyll fluorescence readings revealed changes in transient NPQ that may indicate delayed CBBC activation in *pgdh3* mutants. Consequentially, we analyzed CO_2_ fixation by gas exchange measurements.

**Figure 5:**
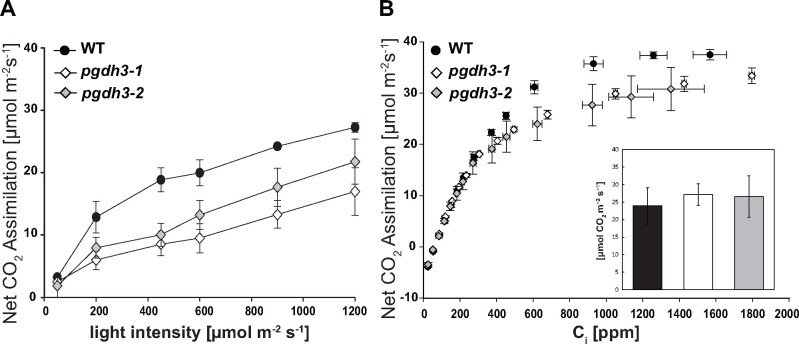
Gas exchange in WT and *pgdh3* reveal lower CO_2_ fixation rates in mutants. A, Light response curve (50–1,200-*μ*mol photons m^−2^ s^−1^) at 1000 ppm CO_2_. B, A/C_i_ curve recorded at a light intensity of 1,200-*μ*mol photons m^−2^ s^−1^. Inlet: no changes in Rubisco activity were found among genotypes (for all experiments ±se, *n = *3 per genotype.

Following the guidelines for gas exchange measurements in Arabidopsis (Sharkey, 2019) plant growth conditions were changed to short day conditions (150-*µ*mol photons m^−2^ s^−1^ illumination in 8-h/16-h day–night cycle) to accumulate sufficient plant biomass. Nevertheless, *pgdh3* loss-of-function mutants grew indistinguishable from WT controls (Supplemental Figure 5A). Initially, light curves at 1,000 ppm CO_2_ were recorded (Figure 5A). Starting at 200-*µ*mol photons m^−2^ s^−1^ and above, both *pgdh3* lines revealed a reduced CO_2_ net assimilation rate by about 25%. Next, A/C_i_ curves were recorded at saturating light conditions (1,200-*µ*mol photons m^−2^ s^−1^) with increasing C_i_ levels ranging from 10 to 1,800 ppm (Figure 5B). At internal CO_2_ levels below 300 ppm, the net CO_2_ assimilation rate was comparable between mutant and WT plants. Once internal CO_2_ surpassed this level, we observed a decrease in CO_2_ net assimilation rate in *pgdh3-1* and *pgdh3-2* lines. A Rubisco activity assay on the same leaf tissue did not indicate any changes from WT activity (inlet Figure 5B). Therefore, changes observed in the loss of function mutants are not caused by a lack of Rubisco enzyme or a direct inhibition of Rubisco activity. The A/C_i_ experiment was repeated three times with similar results (Supplemental Figure 5, B and C).

### Subcellular metabolite analysis revealed changes in the pool sizes of primary metabolites and cofactors in pgdh3 loss-of-function mutants

To elucidate the role of PGDH3 in shaping photosynthesis and metabolism, we analyzed the total and subcellular content of a subset of primary metabolites and cofactors. To this end, nonaqueous fractionation (NAF) on midday harvested leaf tissue from 3-week-old plants (16-h/8-h day–night cycle, 150-*µ*mol photons m^−2^ s^−1^) was performed. Metabolites and cofactors were quantified in total and subcellular fractions by chromatography-coupled mass spectrometry.

**Figure 6:**
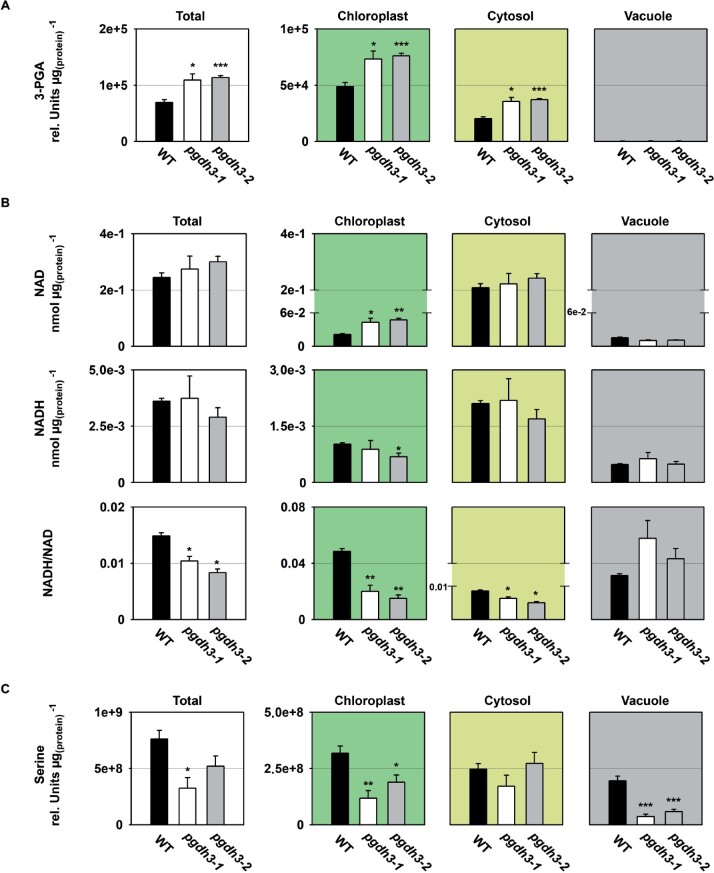
Cellular and subcellular content of PPSB-related metabolites and cofactors. The cellular and subcellular content of (A) 3-PGA, (B) NAD(H), and (C) serine is shown in WT and *pgdh3* mutants. Data presented are means (±se, *n *=* *4). Asterisks indicate significantly different values between WT and *pgdh3* mutant lines by the Student’s *t* test (*p < 0.05; **p < 0.01; ***p < 0.001).

Initially, we focused on metabolites and cofactors related to the enzymatic reactions of the phosphorylated pathway of serine biosynthesis (PPSB; Figure 6, A–C). We found that 3-PGA, the substrate of PGDH enzymes, accumulated significantly in leaves of *pgdh3* mutants (Figure 6A), indicating that the diurnal flux through the PGDH3-mediated serine biosynthesis pathway is relatively high in C_3_ plants. Subcellular metabolite analysis revealed that the content of 3-PGA increased in chloroplasts and the cytosol of *pgdh3* mutants (Figure 6A). As the PGDH3 enzyme is specifically localized in chloroplasts (Figure 1; Benstein et al., 2013; Toujani et al., 2013), the increase of cytosolic 3-PGA in the mutants is most likely the consequence of an elevated export of 3-PGA, either directly, mediated by the triose phosphate/phosphate translocator, or indirectly, as part of the triose phosphate/3-phosphoglycerate shuttle (Flügge et al., 2011; Weber and Linka, 2011).

Next, we measured the size and redox state of the NAD(H) pool in total and subcellular fractions of the plants (Figure 6B). In line with the substantial accumulation of 3-PGA, the NAD(H) pool remained more in the oxidized state in total extracts of *pgdh3* mutants. Interestingly, the NADH/NAD ratio was not only lower in chloroplasts of *pgdh3* mutants but also significantly decreased in the cytosol. Thus, chloroplast localized PGDH3 seem to be involved in the transfer of photosynthetically produced redox energy from the chloroplast into the cytosol. It can be assumed that this export is at least to some degree facilitated by the NAD-specific malate valve.

In contrast to the substantial changes in the total 3-PGA content and the total NAD(H) pool, the total serine content was significantly reduced only in *pgdh3-1* (Figure 6C). The mild effects of *PGDH3* loss on the total serine concentration can be explained by the relatively large pool of photorespiratory serine produced in mesophyll cells and the presence of the other PGDH enzymes in heterotrophic tissue (Ros et al., 2014), which most likely mask changes occurring in *pgdh3* mutants. This conclusion was supported by the analysis of the subcellular serine concentrations, which revealed significantly lower serine contents in chloroplasts and the vacuole of *pgdh3* mutants, while the cytosolic serine content was not altered.

**Figure 7:**
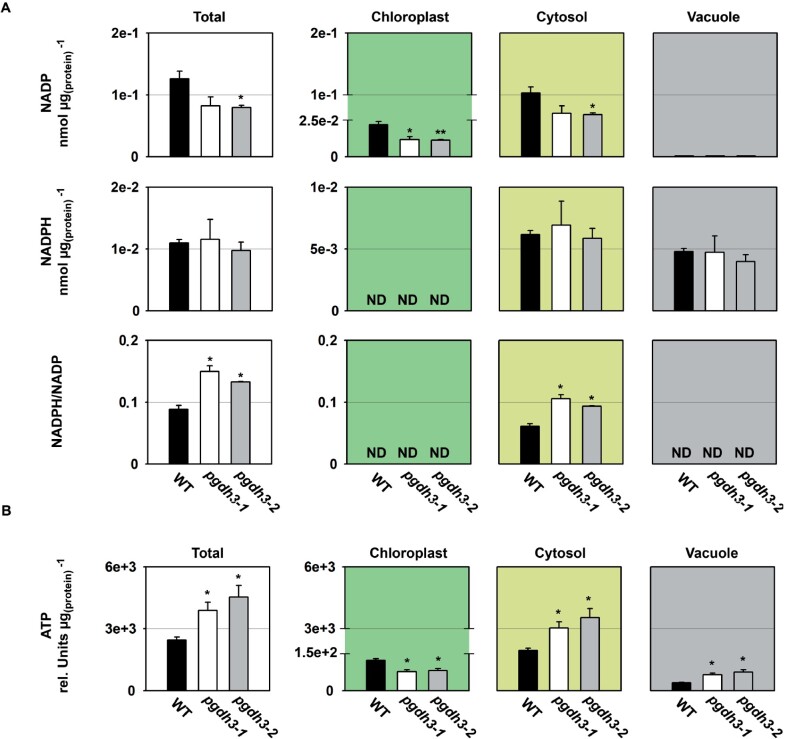
Cellular and subcellular content of NADP(H) and ATP. The cellular and subcellular content of (A) NADP(H) and (B) ATP is shown in WT and *pgdh3* mutants. Data presented are means (±se, *n *=* *4). Asterisks indicate significantly different values between WT and *pgdh3* mutant lines by the Student’s *t* test (*p < 0.05; **p < 0.01). ND = not detectable.

As the lack of *PGDH3* seemed to alter CBBC activation, we next determined the steady-state content and redox state of the NADP(H) pool in total and subcellular fractions (Figure 7A). We found that in our growth conditions around 10%–20% of the total cellular NADP(H) pool remained in the reduced state, which is in agreement with values previously reported for Arabidopsis leaf tissue (Lernmark and Gardestrom, 1994; Beeler et al., 2014). Interestingly, the total content of NADPH was not different in *pgdh3* mutants, while the content of NADP tended to be lower. Similarly, subcellular analysis revealed no changes of the cytosolic and vacuolar NADPH pool, but the content of NADP in chloroplasts and the cytosol significantly decreased. The content of NADPH in chloroplasts and the vacuolar NADP level could not be reliably determined by NAF as their pool size was too small for a trustworthy calculation of the subcellular concentration (Klie et al., 2011). However, by calculating the NADPH/NADP ratio at cellular and subcellular level, we found that the total and cytosolic NADP(H) pool shifted towards a more reduced state in *pgdh3* mutants.

To further investigate the influence of *PGDH3* loss on the energy state of the plant, we analyzed the ATP content in total and subcellular fractions of mutant and WT plants (Figure 7B). We found that the cellular ATP content was significantly higher in *pgdh3* mutants. Interestingly, this increase originated from a higher ATP content in the cytosol and the vacuole, because the ATP content in chloroplasts was lower in the mutant lines compared to the WT.

**Figure 8:**
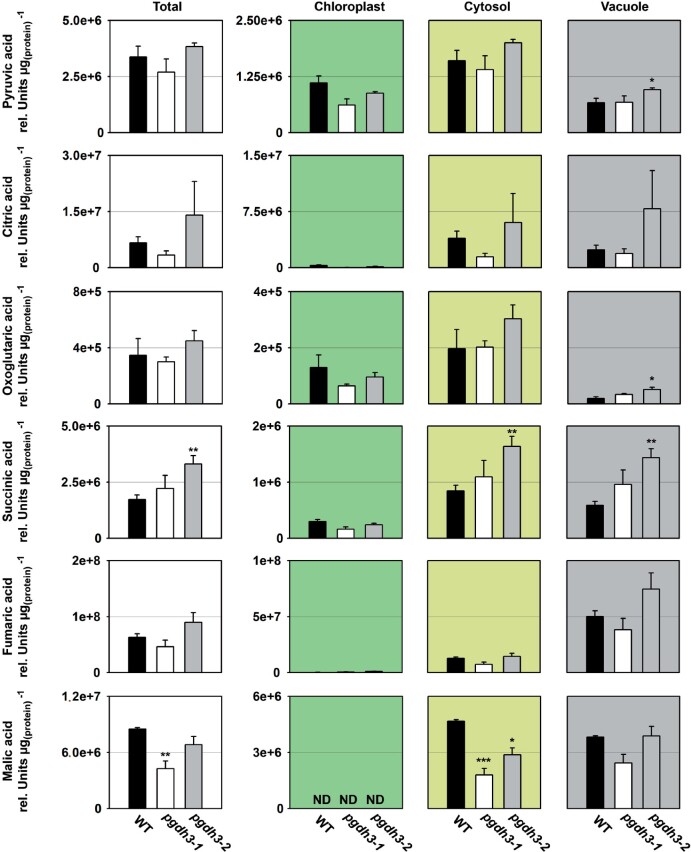
Cellular and subcellular content of organic acids. The cellular and subcellular content of pyruvic acid, citric acid, oxoglutaric acid, succinic acid, fumaric acid, and malic acid are shown in WT and *pgdh3* mutants. Data presented are means (±se, *n *=* *4). Asterisks indicate significantly different values between WT and *pgdh3* mutant lines by the Student’s *t* test (*p < 0.05; **p < 0.01, ***p < 0.001). ND = not detectable.

Next, we determined the cellular and subcellular content of primary metabolites, such as organic acids, amino acids, nitrogen-containing compounds, carbohydrates, and sugar-phosphates (Figure 8 and  Table 2).

**Table 2:** Cellular and subcellular content of primary metabolites

Rel. units µg^−1^ protein	Total			Chloroplast			Cytosol			Vacuole		
Amino acids	WT	*pgdh3-1*	*pgdh3-2*	WT	*pgdh3-1*	*pgdh3-2*	WT	*pgdh3-1*	*pgdh3-2*	WT	*pgdh3-1*	*pgdh3-2*
Glycine	1.9E + 08	1.7E + 08	1.9E + 08	5.2E + 07	**1.6E + 07**	**1.8E + 07**	9.4E + 07	7.6E + 07	8.5E + 07	4.8E + 07	7.5E + 07	**8.5E + 07**
Alanine	4.2E + 08	3.1E + 08	4.3E + 08	1.2E + 08	**5.0E + 07**	**7.0E + 07**	2.2E + 08	1.8E + 08	2.5E + 08	8.1E + 07	8.1E + 07	**1.1E + 08**
Glutamate	1.7E + 08	**8.2E + 07**	1.4E + 08	8.4E + 07	**2.7E + 07**	4.6E + 07	7.9E + 07	4.6E + 07	7.9E + 07	1.2E + 07	8.8E + 06	1.5E + 07
Glutamine	2.5E + 08	**7.8E + 07**	1.5E + 08	1.2E + 08	**4.2E + 07**	7.8E + 07	5.1E + 07	2.8E + 07	5.2E + 07	7.8E + 07	**8.5E + 06**	**1.6E + 07**
Aspartate	9.1E + 07	7.3E + 07	1.6E + 08	2.7E + 07	**1.1E + 07**	2.2E + 07	5.7E + 07	5.2E + 07	1.1E + 08	6.6E + 06	1.0E + 07	**2.2E + 07**
Asparagine	9.3E + 06	1.8E + 06	5.1E + 06	4.8E + 06	**7.4E + 05**	2.1E + 06	1.4E + 06	4.7E + 05	1.3E + 06	3.1E + 06	5.9E + 05	1.7E + 06
Threonine	3.4E + 07	**1.8E + 07**	3.0E + 07	1.1E + 07	**2.3E + 06**	**3.8E + 06**	1.7E + 07	1.1E + 07	1.9E + 07	5.6E + 06	4.3E + 06	7.1E + 06
Methionine	2.2E + 06	2.2E + 06	2.9E + 06	1.2E + 06	**5.3E + 05**	6.8E + 05	9.7E + 05	1.3E + 06	**1.7E + 06**	3.8E + 04	**3.7E + 05**	**4.8E + 05**
Valine	5.7E + 07	3.9E + 07	6.2E + 07	2.1E + 07	**7.6E + 06**	**1.2E + 07**	2.7E + 07	2.2E + 07	**3.6E + 07**	9.7E + 06	8.7E + 06	**1.4E + 07**
Isoleucine	1.4E + 07	7.7E + 06	1.3E + 07	4.5E + 06	**1.4E + 06**	**2.4E + 06**	7.1E + 06	4.4E + 06	7.6E + 06	2.1E + 06	1.9E + 06	**3.3E + 06**
Leucine	1.1E + 07	**5.8E + 06**	9.8E + 06	4.4E + 06	**1.1E + 06**	**1.9E + 06**	4.8E + 06	3.3E + 06	5.6E + 06	1.5E + 06	1.4E + 06	**2.3E + 06**
Phenylalanine	6.4E + 06	**2.9E + 06**	4.4E + 06	2.2E + 06	**4.7E + 05**	**7.1E + 05**	3.3E + 06	**1.9E + 06**	2.9E + 06	9.3E + 05	**5.1E + 05**	7.8E + 05
Tyrosine	2.8E + 06	**1.1E + 06**	**1.8E + 06**	9.0E + 05	**1.3E + 05**	**2.2E + 05**	1.6E + 06	**7.2E + 05**	1.2E + 06	3.2E + 05	2.2E + 05	3.6E + 05
Proline	1.4E + 08	9.7E + 07	1.4E + 08	4.1E + 07	**1.5E + 07**	**2.2E + 07**	8.3E + 07	6.8E + 07	1.0E + 08	1.3E + 07	1.4E + 07	2.0E + 07
**N-compounds**												
ß-Alanine	4.4E + 06	**1.7E + 06**	4.6E + 06	1.2E + 06	**1.9E + 05**	**4.9E + 05**	2.3E + 06	**1.1E + 06**	2.8E + 06	8.5E + 05	**5.0E + 05**	**1.3E + 06**
Spermidine	4.1E + 06	2.6E + 06	3.5E + 06	1.3E + 06	**5.3E + 05**	**7.0E + 05**	2.5E + 06	1.8E + 06	2.5E + 06	2.1E + 05	2.2E + 05	**3.0E + 05**
Ornithine	2.5E + 07	**7.4E + 06**	8.0E + 06	1.2E + 07	**3.1E + 06**	**3.3E + 06**	8.6E + 06	3.2E + 06	3.4E + 06	4.1E + 06	1.2E + 06	1.3E + 06
**Carbohydrates**												
Fructose	2.5E + 07	**1.3E + 07**	2.0E + 07	ND	ND	ND	6.9E + 06	**3.5E + 06**	5.5E + 06	1.8E + 07	**9.7E + 06**	1.5E + 07
Glucose	3.8E + 07	**2.1E + 07**	3.2E + 07	ND	ND	ND	1.3E + 07	8.7E + 06	1.3E + 07	2.4E + 07	**1.2E + 07**	1.9E + 07
Fructose-6-P	9.9E + 02	8.9E + 02	**1.4E + 03**	1.8E + 02	1.4E + 02	2.2E + 02	5.0E + 02	3.8E + 02	5.9E + 02	3.1E + 02	3.7E + 02	5.8E + 02
Glucose-6-P	1.4E + 03	2.9E + 03	**2.1E + 03**	7.0E + 02	**1.9E + 03**	**1.3E + 03**	6.0E + 02	1.0E + 03	7.3E + 02	1.3E + 02	3.2E + 02	**2.3E + 02**

Data presented are means of *n *=* *4. Bold values indicate significantly different values between WT and *pgdh3* mutant plants by the Student’s *t* test (*p* < 0.05).

While the content of most organic acids was not altered in the total fraction of *pgdh3* mutant plants, the content of succinic acid increased only in the *pgdh3-2* line and the content of malate decreased in *pgdh3-1* (Figure 8). Subcellular analysis revealed an increase of pyruvic acid and oxoglutaric acid in the vacuole, and succinic acid in the vacuole and the cytosol of *pgdh3-2* plants (Figure 8). However, the most substantial change of an organic acid was observed for malate, which showed significantly lower content in the cytosol of both *pgdh3* mutant lines (Figure 8).

The analysis of amino acids and other nitrogen-containing compounds showed a clear trend towards lower cellular concentrations, although these changes were often only significant for *pgdh3-1* mutant line (Table 2). Subcellular analysis revealed that the content of most of these compounds was significantly lower in chloroplasts and slightly increased or not altered in the other compartments.

The analysis of carbohydrates revealed a trend towards lower cellular concentrations for glucose and fructose and higher cellular concentrations for fructose-6-phosphate and G6P (Table 2). However, these changes were statistically significant only for one of the two *pgdh3* mutant lines. Interestingly, subcellular analysis revealed significant higher levels of G6P in chloroplasts of *pgdh3* mutant plants, while the other carbohydrates were not substantially altered at the subcellular level in these lines (Table 2). Lastly, we quantified leaf starch contents over the course of the day. Although not significant throughout the day, elevated starch level in both *pgdh3* lines were observed at the onset and the end of the light period (Supplemental Figure 6).

Altogether, our data show that PGDH3 contributes not only to the synthesis of serine in Arabidopsis leaves, but also plays a significant role in the transfer of photosynthetically generated redox energy from the chloroplast into the cytosol. The important role of PGDH3 in these processes further leads to changes in the cellular and subcellular concentration of several primary metabolites.

### The physiological role of PGDH3 is especially critical under fluctuating light stress

An important role for the PPSB in plant stress survival and redox balance was hypothesized in the past (Ros et al., 2014). However, experimental evidence for a specific link between abiotic stress and the PPSB remained limited. In nature, plants frequently experience extreme light intensity shifts resulting in spikes in electron flow, and a buildup of reactive intermediates during high light periods (Armbruster et al., 2017).

The *pgdh3* loss of function mutants revealed changes in NPQ level only during the initial low light phases in our short-term fluctuating light experiment (Figure 3E). However, *pgdh3* plants showed consistent PSI acceptor side limitations and increases in the ΔΨ fraction of the pmf (Figure 4, B and C). Increases in ΔΨ have been linked to enhanced PSII recombination rates (Davis et al., 2016). Therefore, we next tested the impact of extended light stress on *pgdh3* mutants by setting up a long-term phenotyping experiment. For the first 2 weeks, all genotypes were grown at 90 *μ*mol photons m^−2^ s^−1^ (16-h/8-h day–night cycle). At the 2-week age mark, plants were split up into the three treatments groups: control light (CL: 90-*μ*mol photons m^−2^ s^−1^), high light (HL: 900-*μ*mol photons m^−2^ s^−1^), or fluctuating light (FL: 1 min 900-*μ*mol photons m^−2^ s^−1^, 4 min 90-*μ*mol photons m^−2^ s^−1^, average light intensity ∼250-*μ*mol photons m^−2^ s^−1^). WT and *pgdh3* lines were phenotyped for 2 weeks using a customized Imaging PAM script (Schneider et al., 2019).

**Figure 9:**
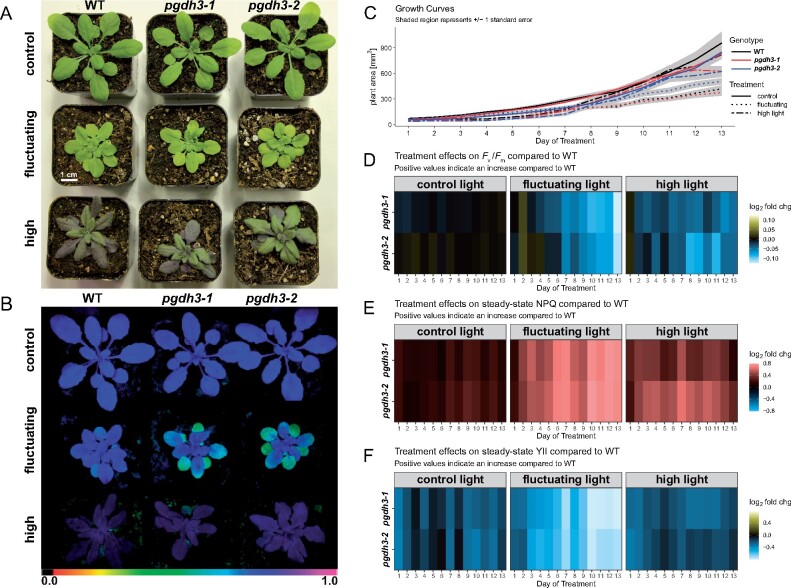
Two-week phenotyping experiment of WT and *pgdh3* mutants subjected to light stress. A, Overall plant appearance changed based on the specific light treatment. B, PSII damage visualized as low *F*_v_/*F*_M_ was found in *pgdh3* mutants only if plants were exposed to fluctuating light. C, Only light dependent but not genotype dependent changes in growth rates were recorded. Heat maps depicting daily WT vs *pgdh3* mutant log_2_ fold changes for (D) *F*_v_/*F*_M_, (E) NPQ, and (F) ΦPSII.

As reported before (Grieco et al., 2012; Thormählen et al., 2017), the light treatments had a strong impact on the overall plant appearance across all three genotypes (Figure 9A). The CL plants were largest in size with a healthy green leaf color. In contrast, FL plants were stunted with paler leaves. HL plants visibly accumulated anthocyanins. Interestingly, when the lines were probed for the maximum quantum yield of PSII (*F*_v_/*F*_M_), only *pgdh3* exposed to FL revealed lower *F*_v_/*F*_M_ than the WT or treatment controls, indicative of PSII damage (Figure 9B). Throughout the duration of this experiment the lower *F*_v_/*F*_M_ did not uniformly affect growth rates in the independent *pgdh3* lines compared to WT (Figure 9C). Given the progressing *F*_v_/*F*_M_ decline in *pgdh3* one may expect that prolonged FL treatments should ultimately culminate in growth rate differences between WT and mutants.

To visualize mutant specific changes from WT in photosynthesis, the daily recorded *F*_v_/*F*_M_, steady-state NPQ, and ΦPSII were plotted as heatmaps (Figure 9, D–F). The same *F*_v_/*F*_M_ difference as noted before (Figure 9B) can be seen in the heatmap, i.e. the two *pgdh3* mutants revealed the most dramatic PSII damage compared to WT under FL (Figure 9D). Initially, HL also decreased *F*_v_/*F*_M_ in *pgdh3*. However, this effect faded over time, indicating the activation of long-term protective mechanisms in all lines.

The importance of PGDH3 under fluctuating light was further emphasized by the collected NPQ data. While we had found that loss of *PGDH3* increased transient NPQ even under standard growth conditions (long day at 90–150 *µ*mol photons m^−2^ s^−1^, Figure 3, C–E) these changes were fairly mild. In contrast, under FL conditions NPQ in *pgdh3* mutants increased much more drastically compared to CL and also HL treatments (Figure 9E). The high NPQ in conjunction with the documented progressing PSII damage (decrease in *F*_v_/*F*_M_) in FL-treated *pgdh3* lines resulted in a dramatic decrease in PSII efficiency ΦPSII (Figure 9F).

In summary, our long-term light stress study of *pgdh3* mutants confirmed that PGDH3 function is required to cope with high energy input during light stress. This is especially important for the C_3_ plant Arabidopsis to sustain fluctuating light conditions, which simulate light energy spikes as they occur frequently in nature.

## Discussion

A gap in knowledge surrounding the chloroplast NAD(H) pool has lingered for a long time (Krause and Heber, 1976; Backhausen et al., 1998). Recent studies showing the importance of the chloroplast NAD-specific malate valve (Beeler et al., 2014; Selinski et al., 2014) and the NAD-dependent steps of fatty acid biosynthesis (Zhao et al., 2018) have re-emphasized the question of which stromal reactions fuel the NADH pool that, for instance, drives the plastid NAD-malate dehydrogenase. Two reactions have been mainly discussed (Zhao et al., 2020): (1) NAD-dependent glyceraldehyde-3-phosphate dehydrogenases (GAPCp) encoded by two loci in Arabidopsis and (2) the PDC. The two GAPCps represent unlikely candidates as their expression is restricted to heterotrophic tissues (Muñoz-Bertomeu et al., 2009). The plastid PDC (PDCp) supplies the enoyl-acyl carrier protein (ACP) reductase (ENR) directly with NADH (Camp and Randall, 1985; Slabas et al., 1986). Therefore, an NADH surplus to drive the significant flux of the NAD-dependent malate valve is unlikely to originate from PDCp activity.

A third so far overlooked option is the PPSB (Ros et al., 2014). Although, activity of its initial reaction step, catalyzed by NAD-dependent PGDH, was found in spinach leaves (Larsson and Albertsson, 1979), the PPSB has drawn little attention as a potential supplier for NADH in illuminated chloroplasts.

In this study, we found high NAD coenzyme specificity over NADP for the three Arabidopsis PGDH proteins (Figure 1, A–C). All isoforms were active under reducing buffer conditions at pH 8.0, showing enzyme activity is feasible throughout the day. PGDH3-GUS signal emerged from mesophyll cells while PGDH1 was more restricted to leaf veins. PGDH2 was barely expressed in leaves (Figure 1D). Immunoblotting and enzyme activity tests using two independent *pgdh3* loss-of-function lines showed roughly a 38%–47% decrease in signal and activity (Figure 2, C and D). Because samples were collected from total leaf extract the exact contribution of PGDH3 to total PGDH activity in mesophyll chloroplasts of Arabidopsis could be higher. When probing the impact that the loss of plastid PGDH isoforms may have on photosynthetic performance, exclusively *pgdh3* mutants revealed higher PsbS-dependent transient NPQ during induction of photosynthesis (Figure 3, C and D). When dark-adapted WT plants become illuminated with nonsaturating actinic light pulses, initially NPQ increases rapidly as ΔpH builds up. However, after ∼1–2 min, once stromal CO_2_ fixation in the CBBC begin to consume ATP and NADPH, ΔpH across the thylakoid membrane decreases and a significant drop in NPQ (and qE) is noticeable (Kalituho et al., 2007; Cardol et al., 2010). This NPQ drop was strongly delayed in *pgdh3* loss of function mutants, which hints to a slower CBBC activation (Figure 3C). Besides these changes during photosynthetic induction, several photosynthetic parameters were also markedly altered under conditions of steady-state photosynthesis (Figure 4). We found a pronounced PSI acceptor side limitation in both mutants. Additionally, under light-limited conditions, the pmf was mostly stored as ΔΨ. In line with this observation, NPQ was not triggered to the same degree in *pgdh3* during slowly performed light curve measurements. Notably, we found no evidence that photosynthetic control via the cytochrome b_6_f complex is affected in mutants.

The quantification of subcellular metabolite pools, especially those linked to the CBBC, provided insights in the connection between the stromal PGDH function and the light reactions in thylakoid membranes. The accumulation of its substrate 3-PGA and lower amounts of the final PPSB product serine in the chloroplast fraction indicate that flux trough the PPSB is higher in illuminated leaf cells than previously assumed (Figure 7, A and C; Samuilov et al., 2018). Furthermore, the general buildup of 3-PGA also hints at a lower CBBC rate, which was also seen in the A/C_i_ curves at ambient and especially elevated CO_2_ concentrations, when a 3-PGA buildup can contribute to triose phosphate utilization limitation (Figure 5B; McClain and Sharkey, 2019). Starch synthesis at the beginning and end of the light period was elevated in *pgdh3* (Supplemental Figure 5), in line with the role of 3-PGA as an allosteric activator of AGPase (Geigenberger, 2011).

Early ^14^C pulse experiments revealed an incomplete labeling of CBBC intermediates, which had plant scientists posit the existence of alternative reaction pathways connected to the CBBC, potentially to adjust CBBC flux according to metabolic requirements (reviewed in Sharkey, 2019). One of these pathways is the proposed G6P shunt, which may be beneficial under high light stress to protect PSI via cyclic electron flow and ATP consumption through the shunt (Sharkey and Weise, 2015). Our spectroscopy and metabolite results raise the question: Is the PPSB another auxiliary CBBC reaction pathway? Furthermore, is the decreased carbon flux into the pathway’s end product, serine, responsible for the effects detected in the light reactions, or is the NAD-dependent oxidation–reduction catalyzed by PGDH3 more critical? A substantial lack of serine is rather unlikely as the cytosolic serine pool was almost unchanged and the total pool was only significantly lower in one line. However, in both loss-of-function lines, impacts on the chloroplast and cellular redox level were observed. PGDH3 seems to contribute substantially to the chloroplast NADH pool. As a result, the stromal NADH/NAD ratio in *pgdh3* lines remained significantly more oxidized (Figure 7B). Interestingly, we found that PGDH3 loss also decreased the cytosolic NADH pool (Figure 7B). Therefore, we conclude that PGDH3 is important for the transfer of redox energy from chloroplast into the cytosol. This process should involve the NAD-MDH-dependent malate valve which operates at a similar NAD(H) *K*_m_ as PGDH3 (An et al., 2016). Support for this idea comes from significantly lower cytosolic malate level in both mutant lines (Figure 8B), indicating that the NADP-dependent malate valve does not compensate effectively for reduced flux through the NAD(H) malate valve. When we measured the activity of each plastid malate dehydrogenase individually, no changes from WT were observed, suggesting that neither malate valve is strongly upregulated in *pgdh3* (Supplemental Figure 7). However, this does not undermine the possibility that the flux through the valves might be different in WT and *pgdh3* lines under *in vivo* conditions. The high level of 3-PGA found in the cytosol hints toward increased triose phosphate chloroplast export rates via TPT. High TPT activity might be another way to balance the stromal redox poise in the absence of PGDH3 (Unal et al., 2020). Elevated triose-phosphate export may in part also explain the changes in the ATP level in chloroplasts and the cytosol of *pgdh3* mutant as the triose-phosphate shuttle is intimately linked with subcellular transport of ATP-bound energy (Heldt and Flügge, 1987). Furthermore, elevated starch synthesis (Supplemental Figure 6) might additionally lower the ATP level in the chloroplast of *pgdh3* mutant plants. Spectroscopic analysis revealed no changes in total pmf and *g*H^+^, indicating that the general capacity of the plastid ATP synthase in *pgdh3* mutants is not impaired (Figure 4C).

Our attempts to probe the NADP(H) pool show significantly less oxidized NADP in the stroma but also in cytosol and total fractions (Figure 8A). We could not resolve the chloroplast reduced coenzyme pool, but cytosolic and total NADPH pool were unchanged. Because of the low NADP level, the mutant NADPH/NADP ratio shifted toward a more reduced state. This indicates that *pgdh3* mutants struggle to resupply oxidize NADP. Efficient photosynthesis relies heavily on continuous supply of oxidized NADP ready to accept electrons from the light-dependent reactions (Kramer and Evans, 2011; Dietz et al., 2016; Alric and Johnson, 2017). Thus, the obtained NADP(H) and NAD(H) ratios could explain the observed PSI acceptor side limitation and the delayed CBBC activation (Figures 4 and 5).

Lastly, decreased chloroplast amino acid level in *pgdh3* plants should be acknowledged as another interesting phenotype. While we cannot explain the phenomenon on the basis of our experiments, it could be discussed as follows: Most amino acids are synthesized in chloroplasts/plastids (Heinig et al., 2013; Hildebrandt et al., 2015). Impairment of amino acid biosynthesis in chloroplasts due to lower ATP levels for glutamine synthesis and thus for primary nitrogen fixation can be excluded as glutamine is not significantly altered (Table 2). In addition, such a scenario should affect the total content of all amino acids and not only the subcellular distribution of some. However, it might be that exchange of amino acids between chloroplast and the cytosol is altered. It remains largely unknown how amino acids are shuttled across the plastid envelope membrane (Weber and Linka, 2011). Only few transport proteins have been functionally characterized (Renné et al., 2003; Pudelski et al., 2010). Thus, the energy state, organic acid content, or other factors altered in chloroplasts of *pgdh3* may influence amino acid transport across the envelope membrane.

**Figure 10:**
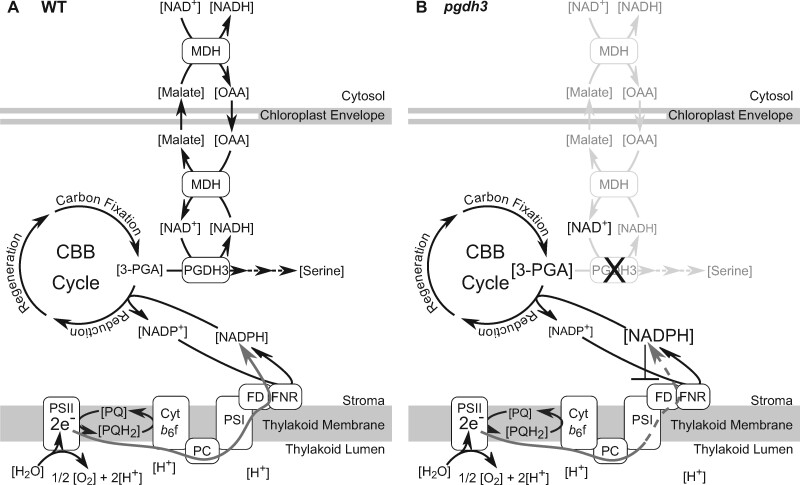
Model of PGDH3’s role in providing an additional stromal electron sink for photosynthesis. A, In WT chloroplasts, PGDH3 facilitates the transfer of electrons from the CBBC intermediate, 3-PGA, to NADH. As a byproduct serine is produced. Electrons can be further transferred to malate by NAD-dependent MDH for export into the cytosol. B, In *pgdh3* mutants, 3-PGA accumulates and less plastid NADH gets produced as an electron sink. Consequently, the CBBC cycle slows down, the NADP(H) pool becomes over reduced, and PSI becomes increasingly acceptor side limited. OAA, oxaloacetate. PSII/I, photosystem II/I. Cyt b_6_f, cytochrome b_6_f complex. PC, plastocyanin. FD, ferredoxin.

To integrate our results within the current framework of photosynthetic electron flow, we drew a model comparing the critical NAD-dependent processes in WT and *pgdh3* mesophyll chloroplasts (Figure 10). In WT, electrons mostly migrate through linear electron flow (LEF) into the NADPH pool (Figure 10A). This is catalyzed by the FNR, a highly specific enzyme that does not except NAD(H) as a cofactor (Piubelli et al., 2000). In other words, the stromal NAD(H) pool cannot be directly reduced by LEF. Most NADPH is used to fuel the CBBC and to a lesser extent the thioredoxin pool (Dietz et al., 2016). The surplus of NADPH is assumed to be exported via the NADP-dependent malate valve. Notably, NADP-MDH loss-of-function mutants are phenotype free. It was suggested that the alternative NAD-dependent malate valve can compensate the loss of the NADP-dependent valve. Indeed, NAD-MDH transcripts accumulate in the absence of NADP-MDH (Selinski and Scheibe, 2014). However, transhydrogenases, which could directly convert NADPH into NADH to enable the suggested bypass, are present only in bacteria (Jackson, 2012). Thus, it was not clear how electrons stored in the NADPH pool could be relayed into NADH to utilize the NAD-dependent valve.

Our findings on PGDH3 suggest that the enzyme indirectly supports NADPH oxidation by diverting electrons from the CBBC into the NADH pool. The reaction serves as an additional electron sink and increases the overall electron storage capacity of the CBBC. Reduced NADH can either fuel fatty acid biosynthesis or become exported via the NAD-dependent malate valve. A loss of PGDH3 activity hampers electron flow into NADH, and thus NADH, malate, and serine levels decline (Figure 10B). NADPH cannot be oxidized as efficiently, resulting in overreduction of the NADP(H) pool. The lack of electron acceptors downstream of ferredoxin results in overreduction of the stroma and increased PSI acceptor side limitation in *pgdh3* mutants. The fact that *pgdh1* and *pgdh2* loss-of-function mutants displayed WT levels of transient NPQ suggests that PGDH3 may have taken on a very specific role related to balancing the stromal redox poise during photosynthesis. Indeed, the co-expression data further support this idea. While *PGDH3* expression appears in a network of photosynthesis-related genes encoding for components of carotenoid biosynthesis, CBBC, PSI, and the NAD(P)H dehydrogenase-like dehydrogenase complex [which receives electrons from ferredoxin rather than NAD(P)H (Laughlin et al., 2019; Schuller et al., 2019)] (Supplemental Figure 8A), *PGDH1* and *PGDH2* are co-expressed with each other but show no apparent links to photosynthesis (Supplemental Figure 8, A and B).

A constant dilemma for plants growing in nature is the rapidly changing energy input. While the light-reactions are very fast and ATP and NADPH production immediately respond to light fluctuations, the light-independent reactions struggle to keep up with the speed of incoming electrons (Nelson and Junge, 2015; Kaiser et al., 2019). Hence, plants utilize various mechanisms to provide electron sinks. Interestingly, PGDH3 is the only PGDH isoform that accumulates (transcript and protein) in mature leaves exposed to fluctuating light (Schneider et al., 2019; Niedermaier et al., 2020). In line with this, our long-term fluctuating light stress data reveal that the reaction catalyzed by PGDH3 yielding reduced NADH may provide a well-needed electron sink to replenish the oxidized NADP pool but also to keep the CBBC balanced and running. In the absence of PGDH3, the need for PSI and PSII protection becomes so high that once NPQ capacities have been exhausted, PSII damage can no longer be avoided (Figure 9). The pronounced PSII damage under fluctuating light stress may in part be linked to the increased ΔΨ in *pgdh3* mutants, a condition known to trigger charge recombination and protein damage in PSII (Davis et al., 2016; Wang and Shikanai, 2019). It will be important to understand why *pgdh3* plants store their pmf mainly as ΔΨ, especially under light-limited conditions (Figure 4C). One possibility is that the shifted stromal redox poise or altered concentrations of other regulatory metabolites impact the activity of thylakoid ion transport proteins in the mutants. In vascular plants, proton transfer into the lumen is usually coupled to fluxes of counter-ions. Using ion-selective electrodes or patch-clamp measurements, significant light-induced transport at least of Cl^−^, Ca^2+^, Mg^2+^, and K^+^ could be demonstrated (Hind et al., 1974; Schönknecht et al., 1988; Enz et al., 1993; Bulychev and Vredenberg, 1999). So far, only a limited number of thylakoid ion channels and ion-proton-antiporters has been identified in thylakoids, among them the bestrophin‐like Cl^−^ channel AtBEST, also called voltage-dependent chloride channel 1 (VCCN1; DuAn et al., 2016; HerdeAn et al., 2016), and thylakoid K^+^/H^+^ antiporter KEA3 (Armbruster et al., 2014; Kunz et al., 2014). An Arabidopsis loss-of-function mutant of AtBEST shows a major shift of pmf partitioning in favor of ΔΨ (DuAn et al., 2016). In plants overexpressing AtBEST/VCCN1, pmf is predominantly stored as ΔpH. This Cl^−^ channel is voltage-gated, but further regulation by other mechanisms cannot be excluded (HerdeAn et al., 2016). The thylakoid K^+^/H^+^ antiporter KEA3 shifts pmf toward lower ΔpH and is regulated via its C-terminus, which carries a regulatory KTN domain (Armbruster et al., 2016). KTN domains bind NAD(P)/NAD(P)H and ATP (Kröning et al., 2007; Roosild et al., 2009). However, whether KEA3 binds nucleotides and is regulated in such a fashion remains unknown. Taken together, our data indicate that thylakoid ion flux is regulated by the stromal redox poise and/or levels of other metabolites, which are altered in *pgdh3* loss of function mutants. Regulation could occur via currently unknown thylakoid ion transport proteins or via VCCN1 or KEA3, of which decreased or increased activity, respectively, could explain the higher ΔΨ as documented in *pgdh3* plants.

## Conclusions

In this study, we aimed to identify enzymes active in photoautotroph chloroplasts that aid in the organelle’s NADH production. Our result show that PGDH3 is active in illuminated chloroplasts, has high co-enzyme specificity towards NAD(H), and discriminates strongly against NADP(H). The study of *pgdh3* mutants establishes the PGDH3-dependent PPSB as an important source of stromal NADH. Therefore, in WT chloroplasts PGDH3 activity provides an alternative electron sink via the CBBC assisting in replenishing the main stromal electron acceptor NADP. The produced NADH can leave the chloroplast via the NAD(H)-specific malate valve. This pathway is important to balance sudden shifts in photon energy input as plants experience it frequently in nature. Stromal NADH levels only decreased in *pgdh3*. Therefore, other enzymes must contribute to the chloroplast NADH supply and need to be investigated in the future. Our data point towards engineering chloroplast NAD(H) levels through their linked enzymes as yet another potentially interesting path to adjust photosynthetic efficiency in the field.

## Materials and methods

### Plant growth

WT Arabidopsis (*Arabidopsis thaliana*) accession Columbia-0 (Col-0) and mutant plants were germinated on half Murashige & Skoog (MS) 1% (w/v) phytoagar plates at pH 5.8 for 7 d, transferred to soil (Sungro Professional Growing Mix #1, Sun Gro Horticulture, Agawam, MA, USA) and grown under 150-*µ*mol photons m^−2^ s^−1^ illumination in 16-h/8-h day–night cycle, temperatures 23°C/21°C (light/dark). Rosettes of 3- to 4-week-old plants were used for all experiments if not stated differently. For short-term fluctuating light experiments the growth light was reduced to 90–100-*µ*mol photons m^−2^ s^−1^ illumination to match the low light phase during the experiment. Light fluctuation was carried out with a MAXI version IMAGING-PAM (IMAG-K7 by Walz, Effeltrich, Germany). As mentioned before, plants for gas exchange measurement were grown in short days (8-h/16-h day–night cycle) at 150-*µ*mol photons m^−2^ s^−1^ illumination.

### Isolation of single and higher order mutants

The homozygous genotype of T-DNA insertion mutant *pgdh3-1* (SM_3_37584) and *pgdh3-2* (GK-877F12) was confirmed using the following PCR primers: for the WT product *PGDH3* fwd gaaggatccatggcgacgtctctgaa, *PGDH3* rev gatgaggtgtggcagtgacac, and for the T-DNA product Spm1 cctatttcagtaagagtgtggggttttgg in the case of *pgdh3-1* and GABI-LB cccatttggacgtgaatgt for *pgdh3-2*. To study contribution of the PsbS protein, *pgdh3-1* was crossed with the previously established *npq4-1* allele, a fast-neutron mutagenized large deletion line (Li et al., 2000). Homozygous F2 double mutant individuals were isolated using a MAXI version IMAGING-PAM (IMAG-K7 by Walz, Effeltrich, Germany) followed by PCR to verify T-DNA insertion in *PGDH3*.

### Generation of overexpression construct and stably transformed Arabidopsis plants

PGDH overexpression constructs for transient enzyme assays in *N. benthamiana* and stable Arabidopsis overexpression plants were generated by amplifying *PGDH1* (cgcgccactagtggatccatgtcagccaccgccg/cccttgctcaccatcccggggagcttgaggaaaacgaactcctcaac), *PGDH2* (ggcgcgccactagtggatccatggcattttcatcttcgtgttcgtccg/cccttgctcaccatcccgggtagtttaagaaacacaaactcttc), and *PGDH3* cgcgccactagtggatccatggcgacgtctctgaatctatc/cccttgctcaccatcccgggtagtttgaggaaaacaaactcttcaatggcagg) from cDNA pools introducing the coding sequences into BamHI/XmaI cut pHygIIUT-MCS-YFP (*pUBQ10::PGDH1-3-YFP*) vector (Kunz et al., 2014) by Gibson cloning. Each construct was transformed into *pgdh3-1* by floral dip (Clough and Bent, 1998). Individual transgenic plants were selected based on their resistance to hygromycin. The presence of the inserted *pUBQ10::PGDH3-YFP* construct was confirmed by PCR. The level of protein expression was determined by confocal microscopy, immunoblotting, and total PGDH activity assay.

### In vitro PGDH assay in plant extracts

Arabidopsis *PGDH* isoforms were transiently expressed in *N. benthamiana* according to an established protocol (Day et al., 2019). Three days after injection, the infiltrated leaves were ground in extraction buffer containing 200-mM tris(hydroxymethyl)aminomethane (tris), pH 8.0; 1-mM Dithiothreitol (DTT); 1-mM Ethylenediaminetetraacetic acid (EDTA); 0.5% (v/v) Triton X-100; and 1% (w/v) polyvinylpolypyrrolidone. The extract was passed through two layers of miracloth to remove debris. The extract was desalted by loading over a column containing 2.5 mL bed volume of Sephadex 50 pre-equilibrated with storage buffer containing 200-mM tris, pH 8.0; 1-mM DTT; and 1-mM EDTA, then centrifuging the column at 1,000*g* for 2 min. The flow through was collected, and the protein concentration was measured using the Bradford assay (Krause and Heber, 1976).

PGDH enzyme activity assays were prepared in a 96-well, clear bottom plate. Ten microliter of leaf protein extract was mixed with 160-*μ*L reaction buffer containing 200-mM tris, pH 8.0; 0.1-mM DTT and 25-mM EDTA, 10-*μ*L 100-mM hydrazine sulfate, and 10-*μ*L cofactor solution containing a varied amount of NAD or NADP. The reactions were initiated by the addition of 10-*μ*L 200-mM 3-phosphoglycerate with a custom-made 96-headed spatula (WSU Technical Services, Pullman, WA, USA). The plate was quickly inserted into a Tecan M200 PRO plate reader (Tecan, Männedorf, Switzerland), which monitored the reaction progression by measuring the absorbance increase at 340 nm. Data were analyzed using Microsoft excel and GraphPad Prism.

### GUS staining

GUS staining using 5-Bromo-4-chloro-3-indolyl-ß-D-glucuronic acid (X-GlcA; Chem Impex Inc., Palm City, FL, USA) was performed at several developmental stages. The *PGDH* promoter reporter lines (Benstein et al., 2013) were either grown on ½ MS plates or in hydroponics. Plant tissue was stained in 100-mM phosphate buffer, pH 7.0 with Triton and 0.01% (w/v) X-GlcA for 24 h at 37°C in the dark (Munekage et al., 2002).

To visualize the blue GUS staining, leaf chlorophyll was extracted using a series of ethanol dilutions spanning from 40–100% (v/v) ethanol.

### Generation of α-PGDH3(1/2) immunoglobulin

The previously described *PGDH3* cDNA without its transit peptide fused to an N-term His-Tag in pET16b (Benstein et al., 2013) was transformed into *Escherichia coli* expression strain BLR 21. Bacteria were grown in Terrific Broth (24 g·L^−1^ yeast extract, 12 g·L^−1^ trypton, 0.4% (v/v) glycerol) to an OD_600_ between 0.6 and 0.8. The cultures were cooled to room temperature then induced with isopropyl b-d-thiogalactopyranoside at a final concentration of 1 mM. The cultures were shaken at room temperature overnight. Bacteria were collected by centrifugation at 3,220*g* for 10 min. The pellet from 400 mL of culture was resuspended in 16-mL lysis buffer containing 50-mM tris, pH 8.0; 300-mM NaCl, 10-mM imidazole, 1-mg·mL^-1^ lysozyme, 10-μg·mL^-1^ RNase, 2-U·mL^−1^ DNase, and 10-mM MgCl_2_. The bacterial suspension was gently rocked at 4°C for 2 h. Insoluble material was removed by centrifugation at 10,000*g* for 10 min at 4°C. The supernatant was mixed with 2-mL bed volume of pre-equilibrated Ni-NTA agarose and gently rocked at 4°C for 1 h. The mixture was transferred to a column. The lysate was removed by centrifugation at 700*g* for 2 min at 4°C. All subsequent washes and eluates were also removed by centrifugation at 700*g* for 2 min at 4°C. The resin was washed three times with 3 mL wash buffer 1 containing 50-mM tris, pH 8.0; 300-mM NaCl; and 20-mM imidazole. The resin was washed two times with 1 mL wash buffer 2 containing 50-mM tris, pH 8.0; 300-mM NaCl; 250-mM imidazole. Protein was eluted from the resin with three applications of 1-mL elution buffer containing 50-mM tris, pH 8.0; 300-mM NaCl; 400-mM imidazole. The eluates were combined and loaded over 5.5-mL bed volume of Sephadex 50 pre-equilibrated with storage buffer (phosphate buffered saline, 1-mM DTT, and 1-mM EDTA). The isolated protein was buffer exchanged by centrifuging the column at 1,000*g* for 3 min. The protein concentration was measured using the Bradford assay. The protein concentration was brought to 1 mg·mL^-1^ and frozen on dry ice for shipment. The PGDH3 antiserum was raised in rabbits (YenZym Antibodies, San Francisco, CA, USA). To check the antibody specificity against all three Arabidopsis PGDH isoforms, the proteins were produced and purified as described above, but at one-fourth the scale.

### Immunoblotting

Arabidopsis tissue was frozen with liquid nitrogen and ground to a fine powder with mortar and pestle. Protein was extracted by mixing with extraction buffer (200-mM tris, pH 8.0; 4% (w/v) sodium dodecyl sulfate) to 0.5 g fresh weight·mL^−1^ and heating at 90°C for 10 min. Insoluble debris was removed by centrifuging at 21,000*g* for 1 min. The supernatant was removed and mixed with equal volume 2× Laemmli buffer. Samples were loaded on an 8% acrylamide gel. Twenty milliampere (mA) were applied through the gel until the dye front ran off the bottom of the gel. Contents of the gel were electroblotted onto nitrocellulose membrane (0.2-*µ*m pore size) with 70 V for 45 min. The blot was blocked for 10 min at room temperature in tris buffered saline with 0.5% (v/v) tween (TBST) plus 5% (w/v) fat-free powdered milk (blocking buffer). The membrane was incubated overnight at 4°C with the anti-AtPGDH3 antiserum diluted in blocking buffer at 1:3,000 while gently rocking at 75 rpm. The blot was rinsed three times for 5 min each with TBST and subsequently incubated with HRP conjugated goat anti-rabbit secondary antibody (Proteintech Cat# SA00001-2) diluted 1:25,000 in TBST for 2 h at room temperature while gently rocking at 75 rpm. The blot was rinsed three times for 20 min each and then developed with Biorad clarity ECL substrates (Cat#1705060) for 5 min. The signal was collected using a Li-Cor C-DiGit Blot Scanner (LI‐COR Biosciences, Lincoln, NE, USA) using the standard sensitivity setting.

### Pigment analysis

For the analyses of xanthophyll conversion, detached leaves from dark-adapted plants were floated on water at a temperature of 20°C. Leaves were illuminated with white light for 15 min at a light intensity of 900-*µ*mol photons m^−2^ s^−1^ and subsequently exposed to darkness for 30 min. Samples were taken in the dark-adapted state, at the end of the illumination period, and after 2, 10, and 30 min of re-darkening. Samples were immediately frozen in liquid N_2_ and stored at −80°C for up to 24 h. Pigments were extracted with 100% acetone and subsequently quantified by reverse phase High Performance Liquid Chromatography (HPLC; Farber et al., 1997).

### Photosynthetic measurements

All experiments were performed at 22°C and 400 ppm CO_2_. Chlorophyll-*a* fluorescence parameters were determined with the fiberoptics version of the DUAL PAM-100 (Walz, Effeltrich, Germany). Leaves were dark-adapted for 30 min prior to the measurement. Then, under light-limited conditions, the light intensity was increased in 150-s intervals. Under light-saturated conditions above 500 *µ*E m^−2^ s^−1^, the light intensity was increased each 60 s. Electron transport rates of PSII (ETRII) were derived from the quantum yield Y(II) of PSII according to (Genty et al., 1989). The fraction of open PSII centers, qL, was determined as described in (Kramer et al., 2004). PSI measurements were performed with the plastocyanin-P_700_ version of the Dual-PAM instrument (Schöttler et al., 2007), which allows the deconvolution of absorbance changes arising from plastocyanin and PSI. Plants were directly taken from the controlled environment chambers and measured without dark adaptation. Again, the light intensity was stepwise increased as described above for the chlorophyll-*a* fluorescence measurements. The fraction of PSI reaction centers limited at the donor side, Y(ND), or the acceptor side, Y(NA), was determined according to (Schreiber and Klughammer, 2016).

The thylakoid membrane conductivity for protons (gH^+^) was used as a measure for ATP synthase activity. It was determined on intact leaves from the decay kinetics of the ECS during a short interval of darkness. Leaves were pre-illuminated for 6 min with saturating light (1,295 *μ*E m^−2^ s^−1^) so that photosynthesis was fully activated and ATP synthase activity was not limited by ATP consumption by the CBBC. The saturating illumination was interrupted by 15-s intervals of darkness, and the rapid first phase of the decay kinetic of the electrochromic shift during the first 250 ms of darkness was fitted with a single exponential decay function. The reciprocal value of the time constant was used as a measure of ATP synthase activity. The maximum amplitude of the ECS during the first phase of its relaxation kinetic was also used as a measure for the total light-induced pmf across the thylakoid membrane (ECS_T_), and pmf partitioning into ΔpH and ΔΨ was resolved by analyzing the slowly relaxing phase of the ECS between 1 and 15 s of darkness as described by (Takizawa et al., 2007). Between 8 and 10 repetitive measurements of the dark-interval relaxation kinetics were averaged to increase the signal-to-noise ratio. After completing the measurements at 1,295 *µ*E m^−2^ s^−1^, the light intensity was decreased to 144 *µ*E m^−2^ s^−1^ and 88 *µ*E m^−2^ s^−1^, respectively, and plants were given another 5 min to adapt to the new light conditions. The redox state of cytochrome-f was determined in parallel to the ECS measurements. Here, the amplitude of the difference transmittance signal between the fully oxidized state in saturating light and the fully reduced state reached within a maximum of 500 ms in darkness was used as a measure of total redox-active cytochrome-f. Finally, to take differences in leaf chlorophyll content into account, the amplitudes of ECS_T_ and the cytochrome-f difference transmittance signal were normalized to the chlorophyll content per leaf area (Rott et al., 2011). All signals were simultaneously measured between 505 and 570 nm wavelength using the KLAS-100 spectrophotometer (Walz, Effletrich, Germany) and deconvoluted as previously described (Rott et al., 2011).

### Fluorescence emission spectra at 77K

Plant leaves were harvested at day light conditions (16-h/8-h day–night cycle) at 150-*μ*mol photons m^−2^ s^−1^, ground in 25-mM 4-(2-hydroxyethyl)-1-piperazineethanesulfonic acid (HEPES) buffer, pH 7.8 and adjusted to 5-mM chlorophyll. The recording of 77K emission spectra was performed on an AVIV 202SF CD spectrometer as described in (Tietz et al., 2020) using an excitation wavelength of 475 nm. Spectra were normalized to the emission maximum of PSII at 680 nm.

### Gas exchange measurements

Photosynthesis measurements were conducted using the LI‐COR 6400XT gas analyzer (LI‐COR Biosciences, Lincoln, NE, USA). Plants used for gas-exchange measurements were grown in short day conditions (8-h/16-h day–night cycle) at 150-*μ*mol photons m^−2^ s^−1^, 400 ppm CO_2_ for 12 weeks to fill the LI‐COR 6400XT leaf fluorescence cuvette. Before photosynthesis measurements were taken on an Arabidopsis leaf, it was allowed to acclimate to 400 ppm CO_2_ and 1,500-*μ*mol photons m^−2^ s^−1^ (saturating irradiance for these leaves) for 1 h or until steady-state photosynthesis rates were reached. Photosynthetic CO_2_ assimilation versus CO_2_ inside the leaf (A/C_i_ curves) was measured on one leaf from three individual plants for each genotype. Each curve started at 400 ppm CO_2_ and decreased to 0 ppm CO_2_ before returning to 400 ppm CO_2_ and subsequently increasing to 2,000 ppm CO_2_. For each CO_2_ point, individual leaves reached steady-state photosynthesis within 3 min on average before measurements were recorded. For data analysis, the leaf area that covered the cuvette was calculated and corrected in the measurements accordingly. Rubisco activity was determined by recording the NADH oxidation rate to NAD^+^ at 340 nm in an enzyme-coupled spectrophotometric assay as described previously (Boyd et al., 2015).

### Fluctuating light experiments and phenotyping

Light stress and fluctuating light experiments were carried out as described earlier (Schneider et al., 2019). In brief, plants were germinated and grown for 2 weeks at 150-*µ*mol photons m^−2^ s^−1^ illumination at room temperature. At the 2-week mark, plants were split into different pools. One group was kept at constant 90, another at 250, and yet another at 900-*µ*mol photons m^−2^ s^−1^ illumination. The last group was grown under fluctuating light cycling back and forth between 1 min highlight at 900 followed by a 4 min period at 90-µmol photons m^−2^ s^−1^ illumination. All light regimes were applied as long day, i.e. 16-h/8-h day–night cycles. Plant performance was tracked on a daily basis using IMAGING-PAM. Data were analyzed and plotted as described earlier (Schneider et al., 2019).

### Confocal microscopy for protein localization

Images were taken on a Leica SP8 Confocal Laser Scanning Microscope equipped with a supercontinuum laser. For PGDH co-localization experiments with YFP and chlorophyll the 514 nm laser was used for excitation. Subsequently, emission was collected with hybrid detectors at 518–565 nm and 627–706 nm for YFP and chlorophyll-*a* fluorescence, respectively.

### NAF of Arabidopsis leaf tissue

NAF of leaf material obtained from WT and *pgdh3* mutant plants was conducted according to (Krueger et al., 2014). In the middle of the light period, ∼5 g fresh weight of Arabidopsis leaf material was harvested and directly frozen in liquid nitrogen. For homogenization, frozen plant material was placed into a pre-cooled 25 mL grinding jar and homogenized in a tissue lyzer for 1 min at 25 Hz. After homogenization, plant material was lyophilized at 0.02 bar and −80°C for 3 d and afterwards stored protected from light and humidity in a desiccator containing silica gel.

For fractionation, the freeze-dried leaf material was re-suspended in 20 mL tetrachloride ethylene (C_2_Cl_4_):heptane (C_7_H_16_) mixture (66:34; v/v) and sonicated for 2 min with 6 × 10 cycles at 65% power by using a sonicator equipped with a micro tip. To remove bigger particles, the sonicated suspension was filtrated through a nylon net with a 20-*µ*m pore size. The flow through was collected in a 50-mL falcon tube and centrifuged for 10 min at 3,200*g* and 4°C. After centrifugation the supernatant was discarded and the remaining pellet was re-suspended in 3-mL C_2_Cl_4_:C_7_H_16_ mixture (66:34; v/v). Aliquots of 10 × 50 µL were taken and dried in a vacuum concentrator for ∼30 min. The dry aliquots were assigned as “total” and used to determine the total metabolite content in the respective plant line. The remaining suspension was loaded on top of a freshly produced density gradient (for details please see Krueger et al., 2014). The loaded gradient was centrifuged for 1 h at 5,000*g* and 4°C in a swing-out rotor. After centrifugation, four compartment enriched fractions (F1–F4) were taken and transferred into 50-mL falcon tubes using Pasteur pipettes. Three volumes of C_7_H_16_ were added to each fraction, and the suspension was centrifuged for 10 min at 3,200*g* and 4°C in a swing-out rotor. The supernatant was discarded and the remaining pellet was re-suspended in 5-mL C_7_H_16_. Aliquots of 10 × 500 µL of the suspension were transferred into 2-mL reaction tubes and dried in a vacuum concentrator. After drying the aliquots were stored in a vacuum desiccator containing silica gel protected from light and humidity. The quality of the fractionation was tested by the analysis of compartment-specific marker enzyme distribution or marker molecule abundance within the different gradient fractions (for details please see Krueger et al., 2014).

### Analysis of metabolite abundance in fractionated Arabidopsis leaf material

Three different mass spectrometry methods, namely liquid chromatography–mass spectrometry (LC-MS), ion chromatography Mass Spectrometry (IC-MS), and gas chromatography–mass spectrometry (GC-MS), were used to analyze the metabolite abundance in fractionated and nonfractionated leaf material of WT and *pgdh3* mutant plants.

For the analysis of NAD(H) and NADP(H) cofactor abundance a LC–MS-based method previously published by Lu et al. (2018) was used with minor modifications. Aliquots of dried fractionated or nonfractionated plant material were extracted with 250 *µ*L of an acetonitrile: methanol: water (40:40:20; v/v) mixture acidified with 0.1 M formic acid. After incubation for 3 min on ice, 21 *µ*L of 15% NH_4_HCO_3_ were added. The neutralized extract was centrifuged for 15 min at 16,000*g* and 4°C. For LC–MS analysis, 2 *µ*L of the supernatant was injected on a BEH amide column (150 mm × 2.1 mm, particle size 1.7 *µ*m). Compounds were eluted from the column by using a gradient between solvent A (95:5, water: acetonitrile, 20-mM NH_4_CH_3_CO_2_, 20 mM, pH 9.4) and solvent B (acetonitrile). The gradient was 0 min, 90% B; 2 min, 90% B; 5 min, 50% B; 11 min, 0% B; 13.5 min, 0% B; 15 min, 90% B; 20 min, 90% B, with a total running time of 20 min and a flow rate of 150 *µ*L min^−1^. The eluate was injected into a Q Exactive HF mass spectrometer, which was operated in negative ion mode scanning *m/z* 400–800 with a resolution of 140,000 at *m/z* 200. Other MS parameters were aux gas flow rate 10 (arbitrary units), sweep gas flow rate 1 (arbitrary units), spray voltage 3 kV, capillary temperature 300°C, S-lens RF level 65, AGC target 3E6, and maximum injection time 500 ms.

For the analysis of ATP, 3 PGA, PEP, and sugar phosphates 150 *µ*L of the extract was dried in a vacuum concentrator and resolved in 80-*µ*L H_2_O. The samples were centrifuged for 10 min at 16,000*g* and 4°C to remove unsolved particles. The IC–MS analysis was performed according to a previously published method (Schwaiger et al., 2017). In brief, 5 *µ*L of the supernatant was injected on a Dionex IonPac AS11-HC column (2 mm × 250 mm, 4-*μ*m particle size, Thermo Scientific). The column temperature was held at 30°C, while the auto sampler was set to 6°C. A potassium hydroxide (KOH) gradient was generated by the eluent generator using a potassium hydroxide cartridge that was supplied with deionized water. The metabolite separation was carried at a flow rate of 380 *µ*L·min^−1^, applying the following gradient: 0–5 min, 10 − 25-mM KOH; 5–21 min, 25 − 35-mM KOH; 21–25 min, 35–100-mM KOH, 25–28 min, 100-mM KOH, 28–32 min, 100–10-mM  KOH. The column was re-equilibrated at 10 mM for 6 min.

The eluting metabolites were detected in negative ion mode using ESI multi-reaction monitoring (MRM) on a Xevo TQ (Waters) triple quadrupole mass spectrometer applying the following settings: capillary voltage 2.5 kV, desolvation temperature 550°C, desolvation gas flow 800 L h^−1^, collision cell gas flow 0.15 mL min^−1^. All peaks were validated using two MRM transitions: one for quantification of the compound, while the second MRM transition was used for qualification of the identity of the compound. The settings for the MRM transitions are given in Supplemental Table 1. Data analysis and peak integration were performed using the TargetLynx Software (Waters).

The content of organic acids and amino acids in fractionated and nonfractionated plant material was determined by GC–MS analysis. Aliquots of dried material were extracted using Methyl tert-butyl ether (MTBE) as previously described (Krueger et al., 2014). The analysis of metabolites samples was performed using a GC–MS (Q-Exactive GC-Orbitrap, Thermo Fisher Scientific). For this purpose, metabolites were derivatized using a two-step procedure starting with methoxyamination (methoxyamine hydrochlorid, Sigma) followed by a trimethyl-silylation using *N*-methyl-*N*-trimethylsilyl-trifluoracetamid (MSTFA, Macherey-Nagel).

In brief, dried samples were re-suspended in 5 *µ*L of a freshly prepared (20 mg mL^−1^) solution of methoxyamine in pyridine (Sigma) to perform the methoxyamination. These samples were then incubated for 90 min at 40°C on an orbital shaker at 1,500 rpm. In the second step, an additional 45 µL of MSTFA was added, and the samples were incubated for additional 30 min at 40°C and 1,500 rpm. At the end of the derivatization the samples were centrifuged for 10 min at 21,100*g*, and 40 *µ*L of the clear supernatant was transferred to fresh auto sampler vials with conical glass inserts (Chromatographie Zubehoer Trott). For the GC–MS analysis, 1 *µ*L of each sample was injected with a split/splitless injector at 300°C in splitless mode. The carrier gas flow (helium) was set to 2 mL min^−1^ using a 30-m DB-35MS capillary column (0.250 mm diameter and 0.25-*µ*m film thickness, Agilent). The GC temperature program was: 2 min at 85°C, followed by a 15°C min^−1^ ramp to 330°C. At the end of the gradient the temperature was held for additional 6 min at 330°C. The transfer line and source temperature were both set to 280°C. The filament, which was operating at 70 V, was switched on 2 min after the sample was injected. During the whole gradient period the MS was operated in full scan mode covering a mass range *m/z* 70 and 800 with a scan speed of 20 Hz.

For data analysis peak areas of extracted ion chromatograms of monoisotopic peaks of compound-specific fragments [M − e^−^]^+^ were determined using the TraceFinder software (Version 4.2, Thermo Fisher Scientific) with a mass accuracy (<5 ppm).

### Determination of NAD- and NADP-dependent malate dehydrogenase activity in fractionated Arabidopsis leaf material

For the analysis of NAD-dependent malate dehydrogenase activity in WT and *pgdh3* mutant plants, soluble protein extracts were prepared using aliquots of fractionated and nonfractioned plant material and 300-*µ*L ice-cold protein extraction buffer (50-mM HEPES, pH 7.58; 1-mM EDTA; 10% (v/v) glycerol; and 5-mM DTT). Proteins were extracted in a thermal mixer for 10 min at 600 rpm and 4°C. After centrifugation for 10 min at 16,000*g* and 4°C, 10 *µ*L of the supernatant was transferred into a 96-well microtiter plate and mixed with 185-*µ*L assay buffer (90-mM KH_2_PO_4_, 0.05% (v/v) Triton X-100, 5-mM MgCl_2_, 50-*µ*M NADH, pH 7.4). The enzymatic reaction was started by adding 5-*µ*L oxaloacetate (30 mM). The decrease of NADH absorbance was monitored at 340 nm by using a microplate reader.

The activity of NADP-dependent malate dehydrogenase was determined in its reduced and nonreduced state according to (Scheibe and Jacquot, 1983). Therefore, soluble proteins were extracted from fractionated and nonfractionated leaf material of WT and *pgdh3* mutant plants with 300-*µ*L ice-cold extraction buffer (50-mM HEPES, 5-mM MgCl_2_, 10-mM KCl, 2-mM EDTA, pH 7.4). The extracts were incubated in a thermal mixer for 10 min at 600 rpm and 4°C. Afterwards samples were centrifuged for 10 min at 16,000*g* and 4°C to remove insoluble particles. To measure the activity of the NADP-dependent malate dehydrogenase enzyme in its reduced state, 150-µL extract was incubated with 10-*µ*L DTT (160 mM) for 10 min at 26°C. In parallel, 150 *µ*L of the extract was incubated with 10-*µ*L H_2_O for 10 min at 26°C to determine the activity of the nonreduced enzyme. The enzymatic activity was measured in a 96-well microtiter plate by mixing 175-*µ*L assay buffer (100-mM Tris–HCl, 10-mM MgCl_2_, 0.2-mM NADPH, pH 8.0) with 20-*µ*L activated or nonactivated protein extract. The assay was started by adding 5-*µ*L oxaloacetate (40 mM), and the decrease in NADPH absorbance was monitored at 340 nm by using a microplate reader.

### Evaluation of the subcellular distribution of metabolites and enzyme activities

The compartmental distribution of metabolites and enzyme activities in WT and *pgdh3* mutant leaf cells was estimated according to (Riens et al., 1991) using a C version of the compartment calculation program Bestfit (for details, please see Krueger et al., 2014). The subcellular metabolite abundances or enzyme activity was determined by normalizing the calculated percent distribution of metabolites or enzyme activities in the different subcellular compartments to the “total” content or activity of the respective metabolite or enzyme in WT and *pgdh3* mutant plants.

### Accession numbers

*PGDH1* (At4g34200), *PGDH2* (At1g17745), *PGDH3* (At3g19480), *pgdh3-1* mutant (SM_3_37584; Toujani et al., 2013), *pgdh3-2* (GK-877F12; Toujani et al., 2013), *npq4-1* (Li et al., 2000), *npq2-1* aka *aba1-6* CS3772 (Niyogi et al., 1998).

## Supplemental data

The following materials are available in the online version of this article.

**Supplemental Figure S1.** Endogenous PGDH in *N. benthamiana* and expression comparisons for AtPGDH1-3

**Supplemental Figure S2.** Design and testing of α-PGDH3(1/2).

**Supplemental Figure S3.** 77K florescence spectra and isolation of PGDH1 and PGDH3 overexpressor lines.

**Supplemental Figure S4.** Transient NPQ, pigment redox state, and pmf probing of WT vs *pgdh3* mutants.

**Supplemental Figure S5.** Additional bio replicates of A/C_i_ curves for WT vs *pgdh3* mutant plants.

**Supplemental Figure S6.** Transitory starch levels at the start and the end of the day.

**Supplemental Figure S7.** Cellular and subcellular activity of malate dehydrogenase enzymes.

**Supplemental Figure S8.** Coexpression networks of PGDH3, PGDH1, and PGDH2

**Supplemental Table S1.** Settings for the MRM transitions.

## Supplementary Material

kiaa117_Supplementary_Data

## Notes

H.-H.K. and S.K. designed the research, cloned constructs, isolated plant mutants, performed initial experiments, analyzed the data, and wrote the manuscript. R.H. performed most plant experiments and analyzed the data. P.M.D. designed α-PGDH3, carried out all biochemical assays, and assisted in the writing. L.S.L. performed confocal microscopy and plant phenotyping. V.G. and U.A. performed and analyzed the short-term fluctuating light response study. M.K. assisted R.H. in gas exchange studies. P.J. analyzed pigments and performed PSII/PSI spectroscopy studies together with M.A.S, who also determined proton motive force-related parameters. S.K., S.E.Z., and P.G. carried out some pulse-amplitude modulated measurements, GUS, NAF, and metabolite profiling. All authors assisted in editing the manuscript.

The author responsible for distribution of materials integral to the findings presented in this article in accordance with the policy described in the Instructions for Authors (https://academic.oup.com/plphys/pages/general-instructions) is: Hans-Henning Kunz (henning.kunz@wsu.edu).
