# Supplementary material for: Stromal NADH supplied by PHOSPHOGLYCERATE DEHYDROGENASE3 is crucial for photosynthetic performance
Source: Plant Physiol. 2021 Jan 13;186(1):142–67. doi: 10.1093/plphys/kiaa117 (PMC8154072; doi:10.1093/plphys/kiaa117)
Supplement: kiaa117_Supplementary_Data [file kiaa117_supplementary_data.pdf]

Supplemental Figure S1

A

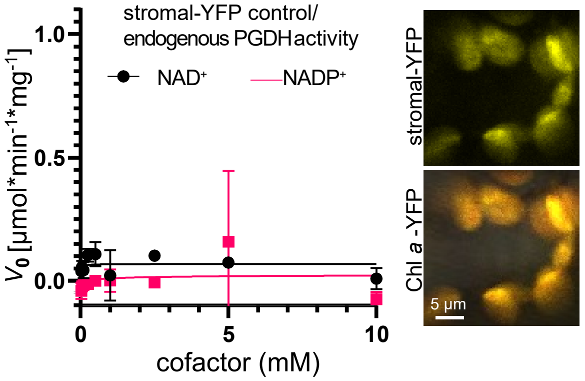

B

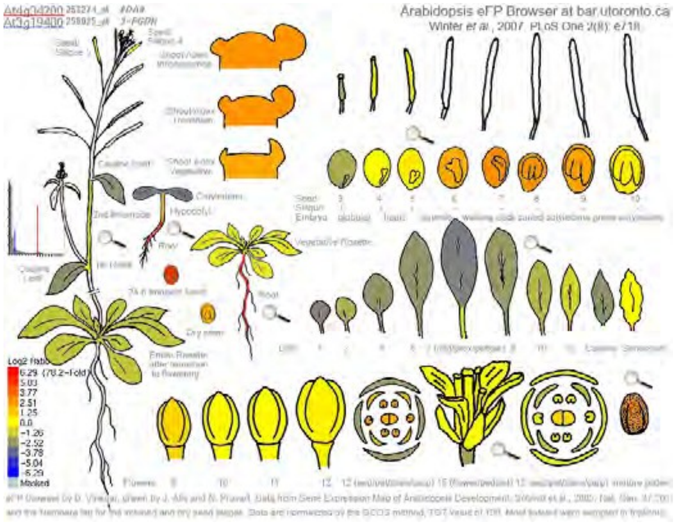

C

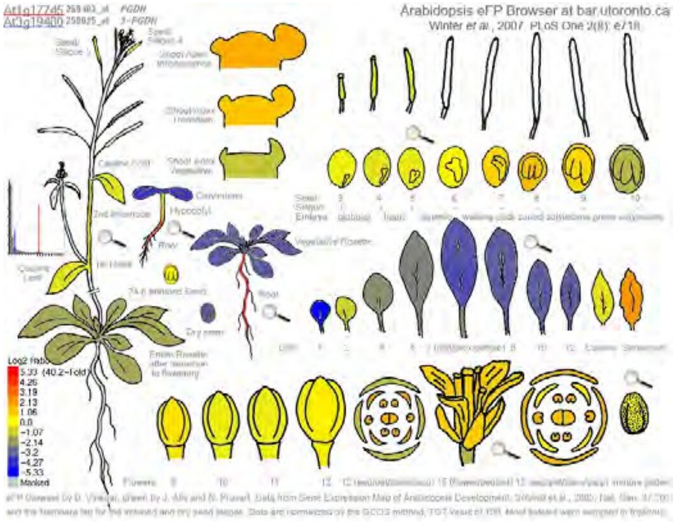

Supplemental Figure S1. A) Endogenous PGDH activity determined in *N. benthamiana* leaves injected with a stromal targeted YFP construct. B) EFP browser gene expression comparison between *PGDH1* and *PGDH3* and C) comparison between *PGDH2* and *PGDH3*.

Supplemental Figure S2

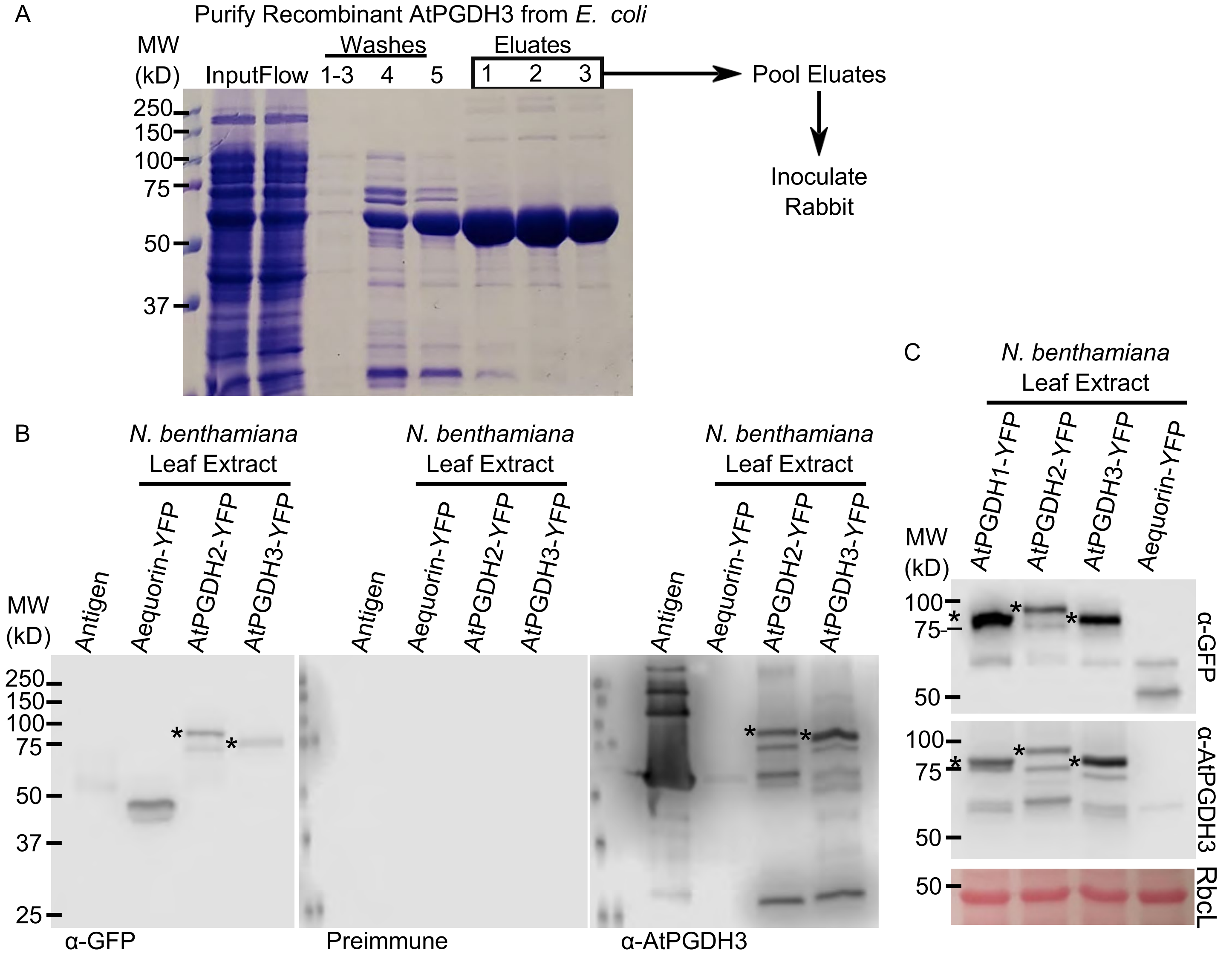

Supplemental Figure S2. Generating an immunoglobulin against AtPGDH3 that recognizes all three *A. thaliana* PGDH isoforms. A) Coomassie stained SDS-PAGE of samples taken during antigen purification from E. coli lysate. Input, cell lysate before mixing with Ni-NTA; Flow, unbound proteins after incubation with Ni-NTA. B) Immunoblots of samples with anti-GFP (α-GFP), preimmune serum and antiserum (α-AtPGDH3) from the inoculated rabbit. Samples include the purified antigen from A and leaf extracts from *N. benthamiana* after transient expression of stromal localized AEQUORIN-YFP, AtPGDH2-YFP, and AtPGDH3-YFP. C) Immunoblots of leaf extracts from *N. benthamiana* after transient expression of AtPGDH1-YFP, AtPGDH2-YFP, AtPGDH3-YFP, and stromal localized AEQUORIN-YFP. Region of ponceau stained membrane occupied by the large subunit of rubisco (RbcL). Asterisks (*) indicate bands corresponding in size to the PGDH-YFP fusion protein.

Supplemental Figure S3

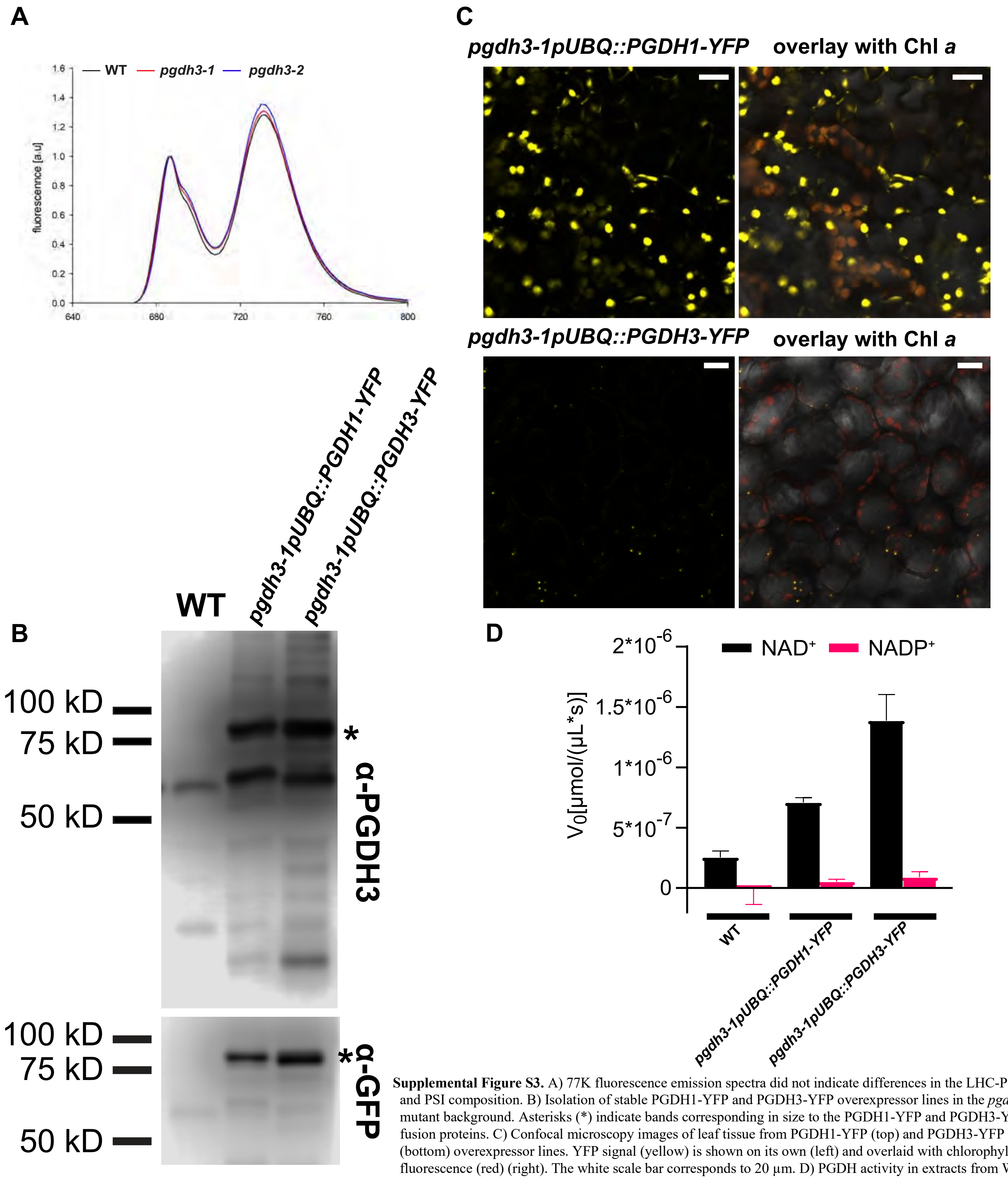

Supplemental Figure S3. A) 77K fluorescence emission spectra did not indicate differences in the LHC-PSII and PSI composition. B) Isolation of stable PGDH1-YFP and PGDH3-YFP overexpressor lines in the *pgdh3-1* mutant background. Asterisks (*) indicate bands corresponding in size to the PGDH1-YFP and PGDH3-YFP fusion proteins. C) Confocal microscopy images of leaf tissue from PGDH1-YFP (top) and PGDH3-YFP (bottom) overexpressor lines. YFP signal (yellow) is shown on its own (left) and overlaid with chlorophyll a fluorescence (red) (right). The white scale bar corresponds to 20 µm. D) PGDH activity in extracts from WT, PGDH1-YFP, and PGDH3-YFP overexpressor lines in the *pgdh3-1* mutant background. Black columns represent activity against NAD while red columns indicate activity against NADP. Activity is shown as µmol cofactor reduced per µL reaction volume per second. Extracts were normalized based on plant tissue fresh weight.

Supplemental Figure S4

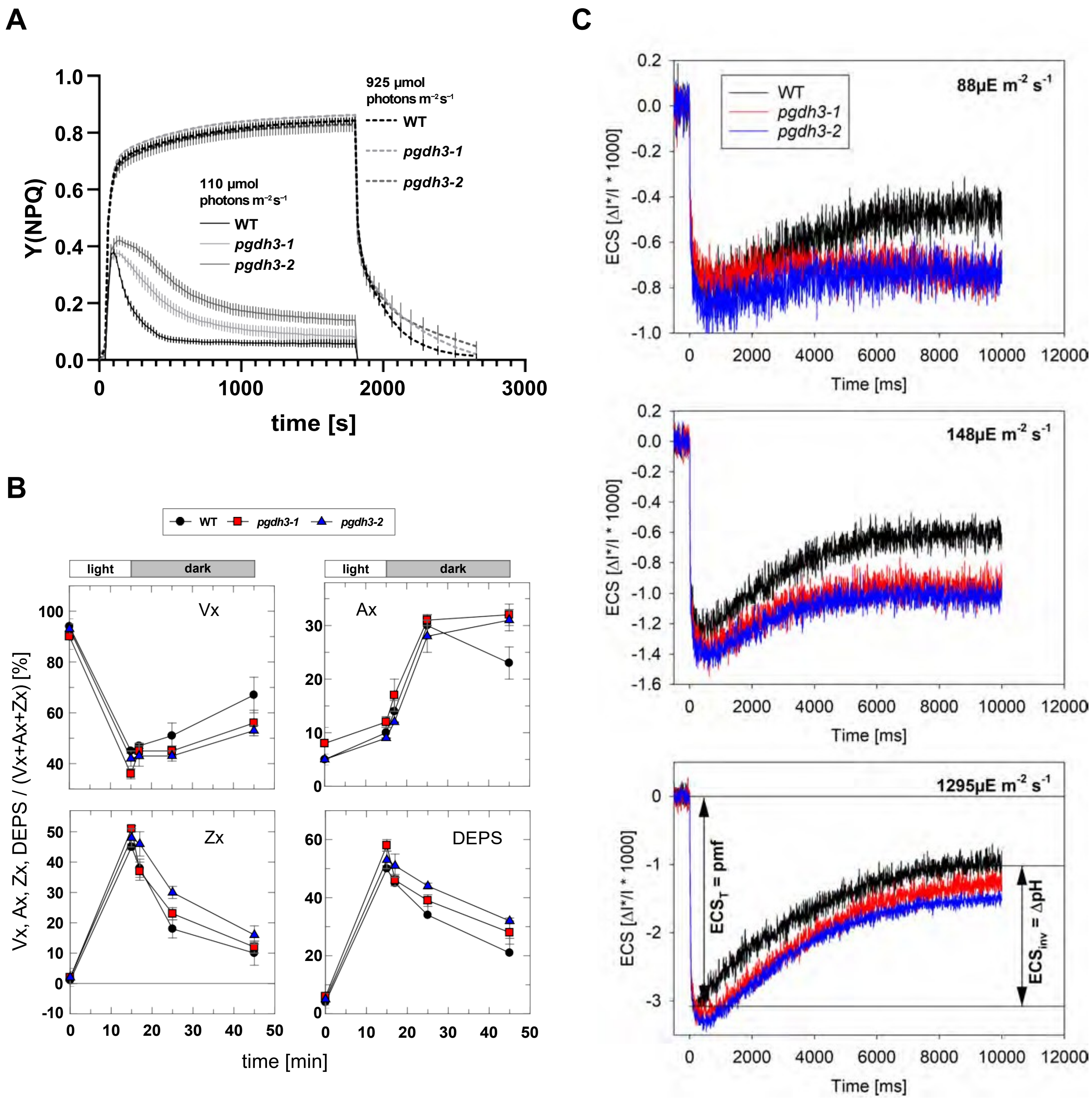

Supplemental Figure S4. A) Recoding of transient NPQ at different light intensities. While under non-saturating light (110 µmol photons m^{-2} s^{-1}) NPQ was strongly elevated in *pgdh3* mutant lines, saturating light conditions (925 µmol photons m^{-2} s^{-1}) abolished these differences. Data represent mean values (± SE) of 6 independent plants per genotype. B) Dark adapted wild type (WT) and *pgdh3* leaves were exposed for 15 min at a light intensity of 900 µmol photons m^{-2} s^{-1}) followed by a 30 min dark period. While no change in de-epoxidation was seen during the light phase, after the light was turned off, both *pgdh3* mutant lines remained in a prolonged de-epoxidation state (DEPS) with Vx level below WT. Data represent mean values (± SD) of 4 independent samples. Exemplary dark interval relaxation kinetics of the ECS signal measured for the wild type and both independent *pgdh3* lines at three different light intensities. At time point zero, the actinic light was switched off, and steady-state photosynthesis was interrupted. The rapid decay of the ECS signal during the first approximately 100 ms in darkness is mainly determined by chloroplast ATP synthase activity, and therefore, the inverse of the lifetime can be used as a measure of ATP synthase activity. The maximum amplitude of the decay is called ECS_T and a measure for the total light-induced pmf across the thylakoid membrane. During the slow inverted phase of the decay kinetics (between several 100 and approximately 10 000 ms in darkness), counter ion movements occur. The amplitude of this inverted phase of the ECS signal (ECS_{inv}) is proportional to the fraction of the pmf stored as ΔpH. Please note the strong decrease of the ECS_{inv} in the *pgdh3* mutants especially under limiting light intensities, indicating that most of the pmf is stored as ΔΨ.

Supplemental Figure S5

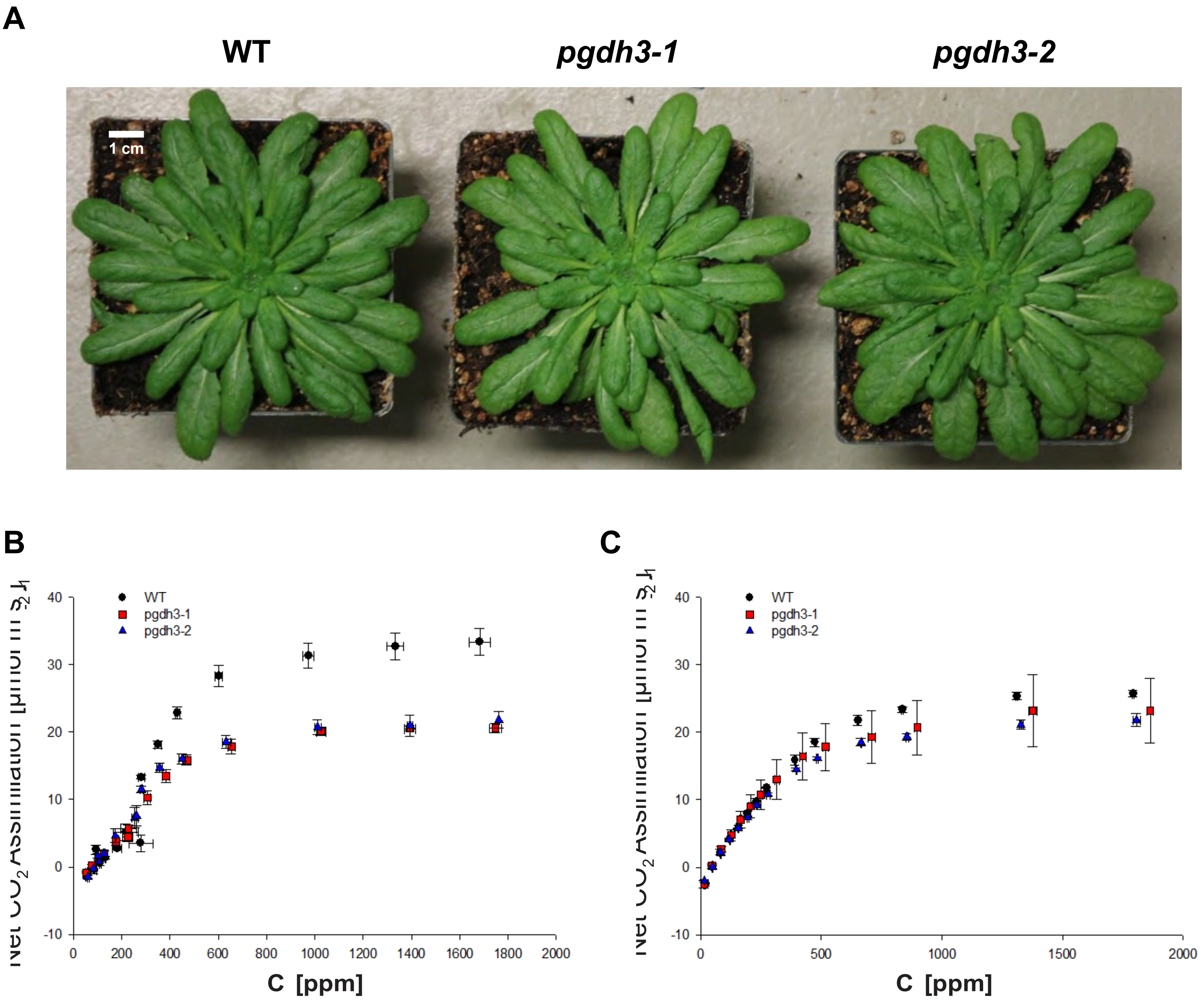

Supplemental Figure S5. A) Two month old short day grown *pgdh3-1* and *pgdh3-2* plants are indistinguishable from WT. B-C) Independent gas exchange measurements on WT and *pgdh3-1* and *pgdh3-2* plants show similar trends as in Fig. 5 (± SE, n=3 per genotype).

Supplemental Figure S6

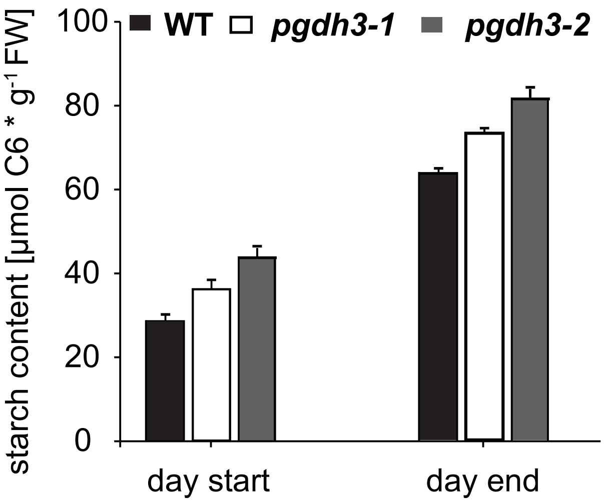

Supplemental Figure S6. Transitory starch level in *pgdh3* mutants are elevated compared to WT at the beginning and the end of the light period.

Supplemental Figure S7

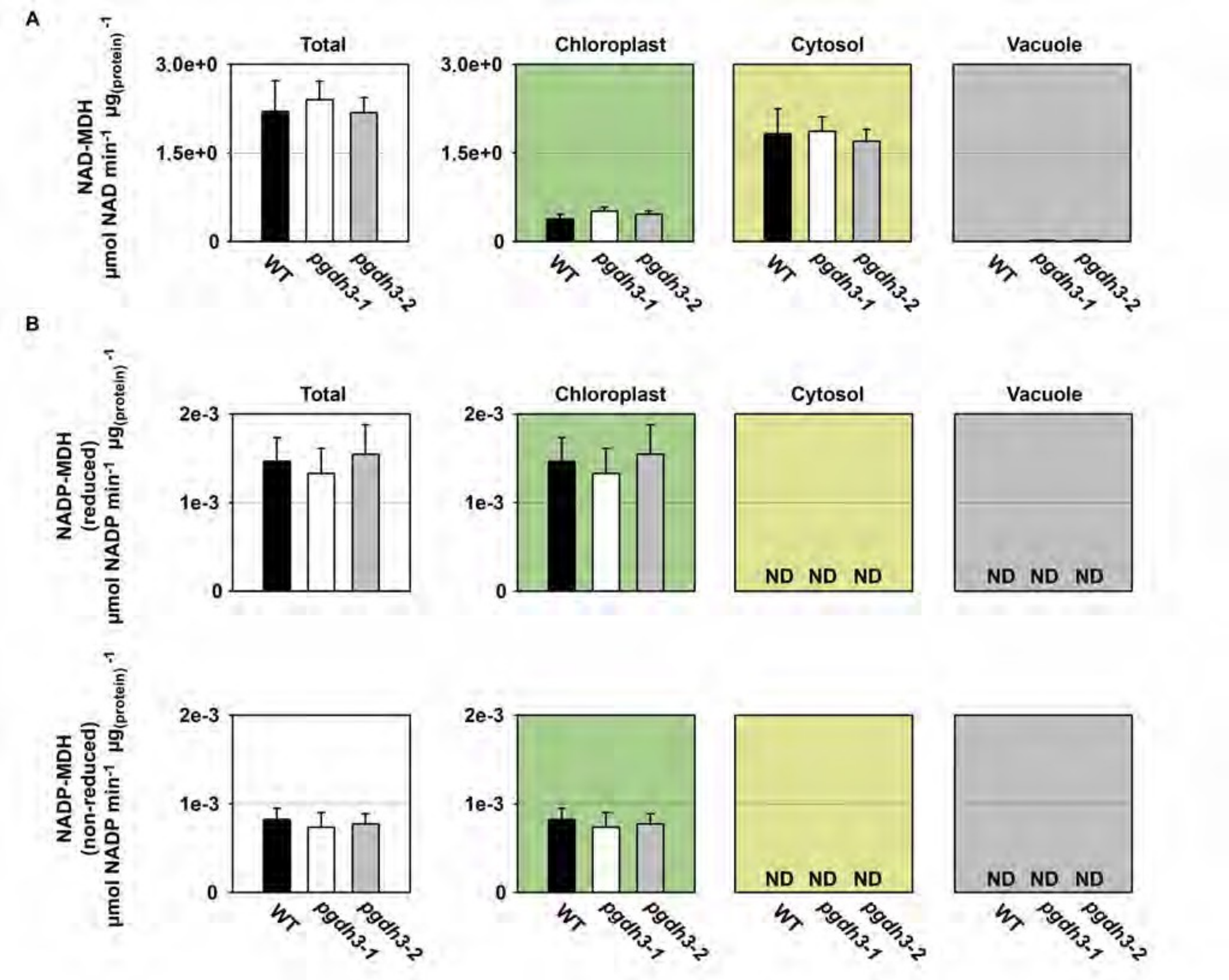

Supplemental Figure S7. Cellular and subcellular activity of malate dehydrogenase enzymes. The cellular and subcellular activity of (A) NAD-dependent malate dehydrogenase and (B) NADP-dependent malate dehydrogenase is shown in wild type (WT) and *pgdh3* mutant plants. The activity of NADP-dependent malate dehydrogenase was determined in non-reduced (non-activated) and reduced (activated) state. Data presented are means ± SE of $n = 4$. ND = not detectable

Supplemental Figure S8

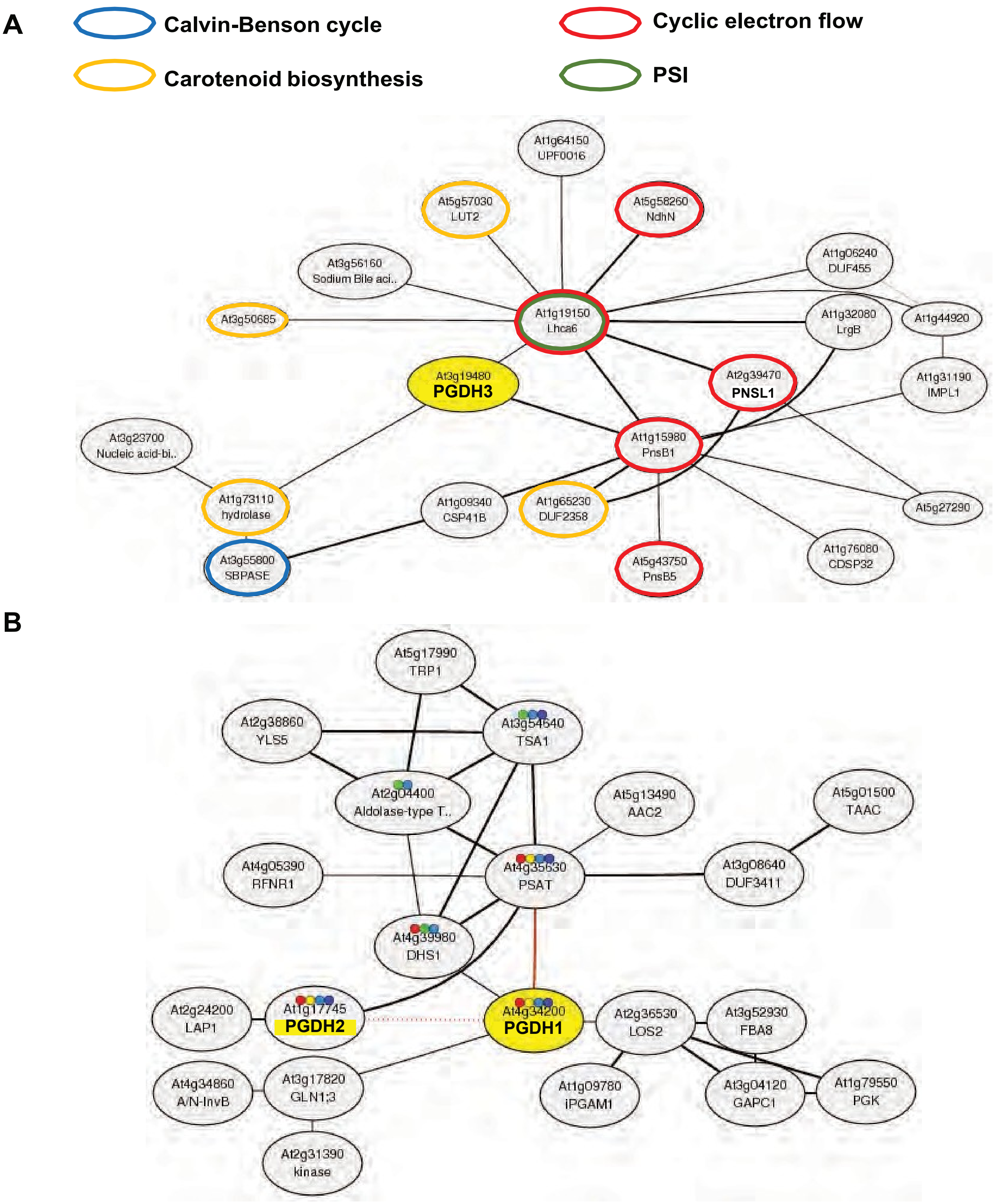

Supplemental Figure S 8. Coexpression network of *PGDH3*, *PGDH1*, and *PGDH2*. Coexpression network of *PGDH3, PGDH1,* and *PGDH2* are strikingly different with only *PGDH3* showing co-expression with components of the light reactions (PSI, NDH) and CO_2 fixation.

Supplemental Table S1. Settings for the MRM transitions

Compound class	Compound	Precursor	Fragment	dwell time	cone V	colision	Function	MRM Type	Ionization mode
glycolysis	3-Phosphoglyceric acid	184,97	96,95	0,057	20	12	9	quantifier	[M-H+]-
glycolysis	3-Phosphoglyceric acid	184,97	78,91	0,057	20	10	9	qualifier	[M-H+]-
glycolysis	Fructose-6P	259,1	78,92	0,061	20	28	13	quantifier	[M-H+]-
glycolysis	Fructose-6P	259,1	96,88	0,061	20	18	13	qualifier	[M-H+]-
glycolysis	Glucose-6P	259,1	78,95	0,061	20	24	14	quantifier	[M-H+]-
glycolysis	Glucose-6P	259,1	96,96	0,061	20	18	14	qualifier	[M-H+]-
glycolysis	Pyruvic acid	86,84	42,95	0,245	18	16	1	quantifier	[M-H+]-
glycolysis	Pyruvic acid	86,84	58,97	0,245	18	8	1	qualifier	[M-H+]-
nucleotide triphosphate	ATP	506,1	158,94	0,066	36	32	35	quantifier	[M-H+]-
nucleotide triphosphate	ATP	506,1	408,04	0,066	36	16	35	qualifier	[M-H+]-
pentosephophate pathway	D-Sedoheptulose-7P	289,07	138,93	0,061	24	26	15	quantifier	[M-H+]-
pentosephophate pathway	D-Sedoheptulose-7P	289,07	78,9	0,061	24	30	15	qualifier	[M-H+]-
pentosephophate pathway	D-Sedoheptulose-7P	289,07	96,92	0,061	24	22	15	qualifier	[M-H+]-
TCA	alpha_Ketoglutaric acid	144,82	56,94	0,061	16	10	6	quantifier	[M-H+]-
TCA	alpha_Ketoglutaric acid	144,82	72,94	0,061	16	14	6	qualifier	[M-H+]-
TCA	Citric acid	190,9	110,92	0,057	20	12	11	quantifier	[M-H+]-
TCA	Citric acid	190,9	86,92	0,057	20	18	11	qualifier	[M-H+]-
TCA	Fumaric acid	114,97	70,96	0,061	18	6	3	quantifier	[M-H+]-
TCA	Fumaric acid	114,97	44,96	0,061	22	20	3	qualifier	[M-H+]-
TCA	Malic acid	132,87	42,94	0,066	18	12	5	quantifier	[M-H+]-
TCA	Malic acid	132,87	70,28	0,066	20	18	5	qualifier	[M-H+]-
TCA	Succinic acid	116,97	72,97	0,066	20	10	4	quantifier	[M-H+]-
TCA	Succinic acid	116,97	98,99	0,066	20	12	4	qualifier	[M-H+]-
